# Identification of new abscisic acid receptor agonists using a wheat cell-free based drug screening system

**DOI:** 10.1038/s41598-018-22538-9

**Published:** 2018-03-09

**Authors:** Keiichirou Nemoto, Makiko Kagawa, Akira Nozawa, Yoshinori Hasegawa, Minoru Hayashi, Kenichiro Imai, Kentaro Tomii, Tatsuya Sawasaki

**Affiliations:** 1 Proteo-Science Center, Ehime University, 3 Bunkyo-cho, Matsuyama, Ehime 790-8577 Japan; 2 Department of Technology Development, Kazusa DNA Research Institute, Kisarazu, Chiba 292-0818 Japan; 3 Department of Materials Science and Biotechnology, Graduate School of Science and Engineering, Ehime University, 3 Bunkyo-cho, Matsuyama, 790-8577 Japan; 4 Artificial Intelligence Research Center (AIRC) and Biotechnology Research Institute for Drug Discovery, National Institute of Advanced Industrial Science and Technology (AIST), 2-4-7 Aomi, Koto Ward, Tokyo 135-0064 Japan

## Abstract

Abscisic acid (ABA) is the main phytohormone involved in abiotic stress response and its adaptation, and is a candidate agrichemical. Consequently, several agonists of ABA have been developed using the yeast two-hybrid system. Here, we describe a novel cell-free-based drug screening approach for the development and validation of ABA receptor agonists. Biochemical validation of this approach between 14 ABA receptors (PYR/PYL/RCARs) and 7 type 2C-A protein phosphatases (PP2CAs) revealed the same interactions as those of previous proteome data, except for nine new interactions. By chemical screening using this approach, we identified two novel ABA receptor agonists, JFA1 (julolidine and fluorine containing ABA receptor activator 1) and JFA2 as its analog. The results of biochemical validation for this approach and biological analysis suggested that JFA1 and JFA2 inhibit seed germination and cotyledon greening of seedlings by activating PYR1 and PYL1, and that JFA2 enhanced drought tolerance without inhibiting root growth by activating not only PYR1 and PYL1 but also PYL5. Thus, our approach was useful for the development of ABA receptor agonists and their validation.

## Introduction

Abscisic acid (ABA) is an important phytohormone for the regulation of complex networks to cope with abiotic stress in plants^1^. The ABA level is regulated through a balance of biosynthesis and metabolism in response to various abiotic stresses, such as drought, salt, and cold^2–4^. ABA is generated through *de novo* synthesis or cleavage of ABA conjugates under abiotic stress^2–4^, and it functions as a trigger for various processes including gene expression for plants to adapt to their environment^5–8^. ABA also plays a key role in plant growth and development under non-stress conditions, such as root growth, stomatal aperture, seed maturation, and dormancy^1,9–12^. Recent studies have revealed that the earliest events involving the ABA signaling pathway occur through ABA-dependent interactions of the core factors consisting of three protein classes: ABA receptors PYR/PYL/RCARs, type 2C-A protein phosphatases (PP2CAs), and subfamily 2 members of SNF1-related protein kinases (SnRK2s)^13,14^. The first step in ABA signaling involves ABA binding to receptors^13,14^. ABA causes a structural change in the ABA receptors and induces the formation of a complex structure with PP2CAs. Formation of the ABA receptor-ABA-PP2CA complex inhibits protein phosphatase activity by masking active sites of PP2CA^15^. SnRK2s are then released following their negative regulation by PP2CAs, causing the phosphorylation of downstream factors to turn on the ABA signals^16,17^. However, some ABA receptors can form complexes with PP2CA even in the absence of ABA^18^. Currently, ABA receptor-mediated inhibition of PP2CAs has been proposed to occur by two mechanisms—ABA-independent or -dependent mechanisms^13,14,18^. Furthermore, the ABA signal module ABA receptor-PP2CA-SnRK2 has been preserved in terrestrial plants, and since ancient times, plants have used ABA-mediated signaling to respond to external stimuli^19,20^.

Previous studies in *Arabidopsis* have shown that all 14 ABA receptors (PYR1 and PYL1 to PYL13) interact with specific PP2CAs in an ABA-dependent or -independent manner and that all ABA receptors are involved in the regulation of ABA signals^14,18,21–23^. However, the commonality and specificity of ABA signals between different abiotic stresses are not well understood because of the functional redundancy of the receptors. Selective ABA receptor agonists that can activate specific ABA receptors would be an effective tool to reveal the connection between specific ABA signals and ABA receptors. Indeed, the ABA receptor PYR1 was identified through a chemical genomics approach using pyrabactin, and it became clear that PYR1 was involved in ABA-dependent seed germination inhibition^14^. Furthermore, in addition to pyrabactin, several ABA receptor agonists for PYR1, PYL1-3, and PYL5, such as quinabactin, have been developed^24^. However, the conventional compound screening is mainly based on phenotypic analysis or the yeast two-hybrid system, and these approaches have some fundamental limitations. Chemical screening methods using living cells often have problems, such as membrane permeability and toxicity of the chemical compounds. Moreover, it is difficult to identify the target molecule of the chemical compound when plant phenotype is used as an indicator in the screening method. In addition, low-throughput is an important issue in chemical screening. Therefore, an *in vitro* technique capable of searching for compounds acting directly on target molecules would be useful for development of chemical compound.

In this study, by using a combination of the wheat cell-free system and the “AlphaScreen” luminescence system, we developed a high sensitivity and specificity as well as high-throughput method to analyze ABA-dependent and -independent interactions and to screen ABA receptor agonists. This method is capable of analyzing the ABA-dependent and -independent interactions under unified conditions without purification of all 14 ABA receptors in *Arabidopsis*. The results of the interaction analysis between the 14 receptors and 7 PP2CAs (ABI1, ABI2, HAB1, HAI1, HAI2, AHG1, and AHG3) exhibited 83.1% commonality with previous proteome data and revealed nine new interactions. Furthermore, interaction analysis within the ABA receptor family revealed 20 new interactions. By applying this method of chemical compound screening based on the interactions between PYR1 and ABI1, we identified one new unique ABA receptor agonist compound JFA1 (julolidine and fluorine containing ABA receptor activator 1) from a chemical library consisting of 9,600 compounds. Thereafter, we newly synthesized a JFA1-like compound, JFA2, which had higher activity than JFA1. JFA1 had high affinity with only PYR1 and PYL1; however, JFA2 had high affinity with PYR1, PYL1, and PYL5. JFA1 and JFA2 suppressed seed germination to the same level; however, JFA2 induced the expression of ABA-inducible genes and enhanced drought tolerance in *Arabidopsis* plants compared to JFA1. These results suggested that our method is useful for the biochemical analysis of ABA receptors and development of an ABA receptor agonist against each receptor, allowing large-scale screening of agonist or antagonist compounds for plant hormones.

## Results

### Development of a wheat cell-free based method to biochemically analyze ABA-dependent protein–protein interactions

**Figure 1:**
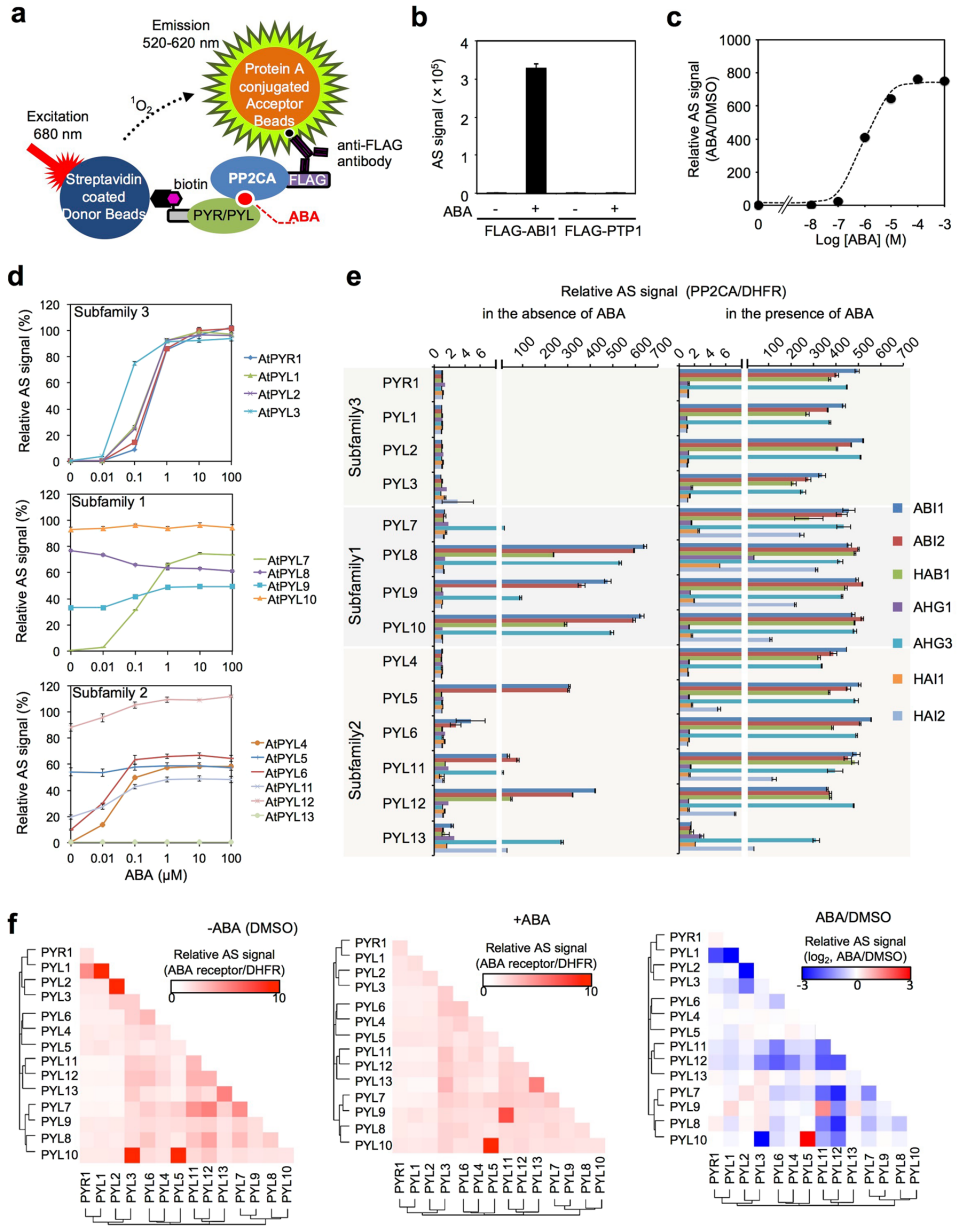
Biochemical characterization of *Arabidopsis* ABA receptors by a wheat cell-free system. (**a**) Principle of receptor-PP2CA interaction analysis by the AlphaScreen. Biotinylated-ABA receptor binds to streptavidin on the donor beads with an extremely specific and high affinity. The protein A-coated acceptor beads are combined with FLAG-PP2CA through the anti-FLAG antibody. ABA receptor-PP2CA complex forms a large complex with two kinds of beads through the antibody and streptavidin. After illumination at 680 nm, the donor beads convert ambient oxygen to singlet oxygen (^1^O_2_), and singlet oxygen is transferred across to activate the acceptor beads and subsequently emits light at 520–620 nm. (**b**) Interaction analysis of biotinylated PYR and FLAG-ABI1. Recombinant biotinylated PYR and FLAG-ABI1 were incubated in the absence (−) or presence (+) of 10 μM ABA. FLAG-PTP1 was used as the negative control. (**c**) ABA-dependent interaction analysis of biotinylated PYR and FLAG-ABI1. The final concentration (M) of ABA is indicated in the graph. Relative AlphaScreen signal (AS) was expressed as a relative value with the signal of DMSO as one. (**d**) Interaction analysis of 14 biotinylated ABA receptors and FLAG-ABI1. Relative AlphaScreen signals were expressed as a relative value with the PYR1-ABI1 interaction signal value in the presence of 100 μM ABA taken as 100%. (**e**) Interaction analysis of 14 biotinylated ABA receptors and FLAG-ABI1, ABI2, HAB1, HAI1, HAI2, AHG1, and AHG3 in the absence (−) or presence (+) of 100 μM ABA. FLAG-DHFR was used as the negative control. Relative AlphaScreen signal was expressed as a relative value with the signal of FLAG-DHFR as one. (**f**) A heat map of the protein–protein interaction AlphaScreen signals between 14 biotinylated ABA receptors and 14 AGIA-ABA receptors. Recombinant ABA receptors were incubated in the absence (left) or presence (middle) of 100 μM ABA. Biotinylated DHFR was used as the negative control. Relative AlphaScreen signal was expressed as a relative value with the signal of biotinylated DHFR as one. The relative change in interaction signal induced by ABA treatment is expressed as log_2_ compared with the signal value of DMSO (right). Error bars represent standard deviations (*n* = 3).

One biochemical property of the ABA receptor is its interaction with the PP2CA in an ABA-dependent manner^13,14^. In our previous studies, we have reported the assay systems that could analyze the biochemical protein–protein interactions using a wheat cell-free system coupled with the AlphaScreen^25–32^. Furthermore, we demonstrated that the phytohormone gibberellin-dependent GID1 receptor-DELLA interaction could be analyzed by using our assay system in a gibberellin concentration-dependent manner^27^. Therefore, using these systems, we attempted to construct an assay system to analyze ABA-dependent interactions between an ABA receptor and PP2CA (Fig. 1a). Protein purification is very time-consuming and requires much work. A major advantage of this system is that it can use non-purified proteins for the assay because translational mixtures, including proteins, produced by the wheat cell-free system can be directly used for developing an assay of protein–protein interactions without protein purification^25–32^. For the construction of an assay system, we chose PYR1 and ABI1, whose biochemical properties are well characterized. N-terminal mono-biotinylated PYR1, FLAG-tagged ABI1, and protein tyrosine phosphatase 1 (PTP1) as a negative control were synthesized by the wheat cell-free system, and we analyzed these interactions in the presence or absence of ABA. The AlphaScreen signal value of PYR1-ABI1 as an index for protein–protein interactions was extremely high in the presence of ABA compared to that in the absence of ABA or PYR1-PTP1 of the negative control (Fig. 1b). Furthermore, the AlphaScreen signal value of PYR1-ABI1 was dramatically increased in an ABA concentration-dependent manner, and this signal value was up to 800-fold higher than in the absence of ABA (Fig. 1c). These results suggested that this assay system could analyze the interactions between ABA receptors and PP2CAs with high sensitivity and specificity without protein purification. To evaluate the effectiveness of this assay system, we next analyzed the biochemical properties of all 14 types of ABA receptors in *Arabidopsis*. All ABA receptors were synthesized by the wheat cell-free system, and it was confirmed that their expression levels were not significantly different (Supplementary Fig. 1a). ABA receptors are largely classified into three subfamilies based on amino acid sequence similarities^13^. Therefore, we analyzed the interaction between ABI1 and the receptors classified in each subfamily. PYR1 was used as a standard for all subfamilies analyzed (Fig. 1d). Interaction analysis showed that PYR1 and PYL1-3 in subfamily 3 interacted with ABI1 in an ABA-concentration-dependent manner (Fig. 1d). In subfamily 1, PYL7 showed an ABA-dependent interaction with ABI1 (Fig. 1d), but other PYLs, such as PYL8-10, interacted with ABI1 in the absence of ABA. Furthermore, in subfamily 2, PYL4 and PYL6 exhibited ABA-dependent interactions with ABI1, and these interactions were promoted at lower concentrations than for PYR1 (Fig. 1d). In contrast, PYL5 and PYL11-12 showed ABA-independent interactions with ABI1. In addition, PYL13 did not interact with ABI1.

Next, we tested whether this assay system could also be applied to other PP2CAs. In *Arabidopsis* genome, nine PP2CAs were found^19,20^, and seven of them were synthesized as N-terminal FLAG-tagged recombinant proteins (Supplementary Fig. 1b) and used for the interaction assay. The results are shown in Fig. 1e. In the absence of ABA, PYL7-10 in subfamily 1, and PYL5, PYL6, and PYL11-13 in subfamily 2 interacted with ABI1, ABI2, HAB1, HAI2, and AHG3 (left panel of Fig. 1e). Furthermore, all 14 receptors interacted with PP2CAs in a specific combination in the presence of ABA (right panel of Fig. 1e). In particular, ABI1, ABI2, HAB1, and AHG3 exhibited interactions with most of the 14 receptors; however, AHG1, HA1, and HAI2 showed selective interactions with the receptors. Finally, 63 interaction pairs of ABA receptors-PP2CAs were detected in this assay. According to the Biological General Repository for Interaction Datasets (BioGRID), the commonality between these results and the previous proteome data was 83.1% (Supplementary Fig. 2). Furthermore, this interaction analysis revealed nine new interaction pairs, PYL8-AHG1, PYL5-11 (excluding PYL8)-AHG3, and PYL11, 12-HAI2.

Another biochemical property of some ABA receptors, such as PYR1 and PYL1-3, is that they form homodimers in the absence of ABA^33–35^. In addition, PYL13 has also been reported to form heterodimers^36^. The homo/hetero dimer interaction surface of the receptor is common to the ABA receptor-PP2CA interaction surface. When ABA binds to a receptor, the monomer receptor dissociates and it becomes possible to form a complex with PP2CA^34,37^. Thus, dimerization of the ABA receptor is thought to be an autoinhibitory mechanism for suppressing the activity of the receptor. Therefore, we conducted an interaction analysis of the ABA receptor families. All ABA receptors were synthesized as N-terminal mono-biotinylated or AGIA-tagged^29^ recombinant proteins. Interaction analysis between ABA receptor families showed that PYL1-3, but not PYR1, formed homodimers and PYL13 formed heterodimers with PYL3 in the absence of ABA (left panel of Fig. 1f, Supplementary Fig. 3). In addition, a total of 24 interaction pairs, including 7 new homodimer interactions and 13 heterodimer interactions were detected in this assay (left panel of Fig. 1f, Supplementary Fig. 3). Among the 24 interaction pairs, 20 interaction pair signals were decreased by ABA treatment (middle and right panels of Fig. 1f, Supplementary Fig. 3). In contrast, another three pairs, PYL13-PYL3, PYL3-PYL3, and PYL13-PYL13, had interaction signals that exhibited little changes with ABA treatment (middle and right panels of Fig. 1f, Supplementary Fig. 3). Interestingly, the interaction of PYL5-PYL10 was dramatically enhanced by ABA, while PYL9-PYL11 was detected only in the presence of ABA (right panel of Fig. 1f, Supplementary Fig. 3). These results suggested that the activity of ABA receptors is controlled by a complex receptor–receptor interaction network. In combination, these results indicated that the cell-free based method could biochemically detect ABA-dependent protein–protein interactions.

### Chemical screening of ABA receptor agonist compounds by using the wheat cell-free based system

**Figure 2:**
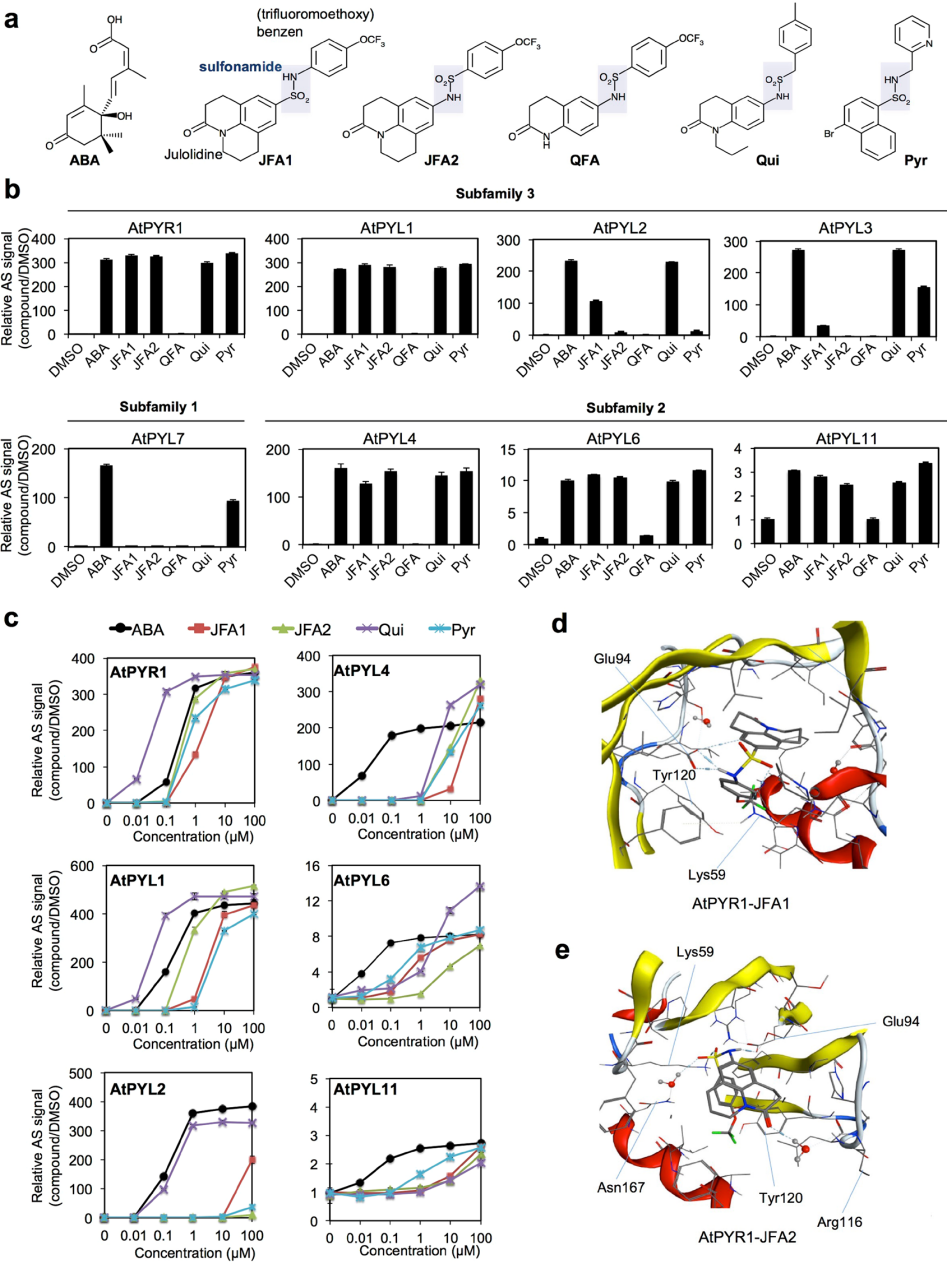
Analysis of the specificity of JFA1 and JFA2 compounds to ABA receptors based on ABA-dependent interaction between ABA receptors and ABI1. (**a**) Structures of ABA and ABA receptor agonist compounds. JFA1 was identified as an agonist in this study, while JFA2 and QFA were identified as JFA1 analogs. Pyrabactin (Pyr) and quinabactin (Qui) were known ABA receptor agonists. (**b**) Interaction analysis of PYR1, PYL1-4, PYL6, PYL7, and PYL11 that interact with ABI1 in an ABA-dependent manner. ABA, JFA1, JFA2, QFA2, Qui, and Pyr were used at a concentration of 100 μM. (**c**) Agonist dose-dependent interaction analysis. Interactions between ABI1 and PYR1, PYL1, PYL2, PYL4, PYL6, or PYL11 were analyzed in the presence of ABA, JFA1, JFA2, Qui, and Pyr. The final concentration (μM) of ABA is indicated in the graph. (**d,e**) Computer docking modeling of the interaction between PYR1 and JFA1 (**d**) or JFA2 (**e**). JFA1/2’s sulfonamide is positioned similarly to ABA’s carboxylate and hydrogen bonds to the amino group of Lys59 and Glu94 in the PYR1 binding pocket. The estimation score of the free energy of binding to the ligand of PYR1-JFA and PYR1-JFA2 were −9.5284 and −10.9391, respectively. AlphaScreen signal was expressed as a relative value with the signal of DMSO (1%) as one, and error bars represent standard deviations (*n* = 3).

By applying the ABA-receptor based interaction analysis system on the wheat cell-free system, we next attempted to identify the ABA receptor agonist and antagonist compounds. To identify the functional compounds, we screened a diverse set of 9,600 synthesized chemicals established by the Drug Discovery Initiative (The University of Tokyo, Japan). Mono-biotinylated PYR1 and FLAG-ABI1 were incubated in the 384-well plate containing 0.6 μM ABA and individual chemicals at 1 µM final concentrations, and the PYR1-ABI1 interaction was analyzed by using the AlphaScreen (Supplementary Fig. 4a). If an agonist or antagonist compound to the receptor was present, the interaction signal of PYR1-ABI1 would increase or decrease, compared to when ABA alone was present. As a result, we identified candidate compounds of 22 agonists and eight antagonists (Supplementary Fig. 4b). Next, we carried out confirmation of the activity of agonist and antagonist candidate compounds and screening of a related compound library (Supplementary Fig. 4c). Finally, we identified 3-oxo-2,3,6,7-tetrahydro-1H,5H-pyrido[3,2,1-ij]quinoline-9-sulfonic acid (4-trifluoromethoxy-phenyl)-amide as an ABA receptor agonist for PYR1, and named it JFA1 (julolidine and fluorine containing ABA receptor activator 1) (Fig. 2a, Supplementary Fig. 4c). Comparing JFA1 with the known agonists pyrabactin (Pyr) and quinabactin (Qui), all the compounds had sulfonamide bonds. Thus, we synthesized two structurally similar analog compounds, JFA2 and QFA (quinoline and fluorine containing ABA receptor activator), based on the structure of JFA1 and quinabactin (Qui), respectively (Fig. 2a). To elucidate the activity of JFA1, JFA2, and QFA for each ABA receptor, we analyzed the agonist activity using eight receptors, PYR1, PYL1-4, PYL6, PYL7, and PYL11, that interacted with ABI1 in an ABA-dependent manner. In the presence of high concentrations of compounds (100 μM), PYR1, PYL1, PYL4, PYL6, and PYL11 were activated by JFA1, JFA2, Pyr, and Qui (Fig. 2b). Conversely, PYL2 was activated by JFA1 and quinabactin, PYL3 was activated by Pyr and Qui, and PYL7 was activated by Pyr only. QFA could not activate any tested ABA receptors. Next, we selected six ABA receptors activated by JFA1 and/or JFA2 and analyzed the reactivity of compounds to their receptors. JFA1 activated PYR1, PYL1, and PYL6 at low concentrations (the relative activity compared with that induced by ABA were 0.18-, 0.05-, and 0.03-fold, respectively, Fig. 2c), but activation of PYL2, PYL4, and PYL11 required high concentrations of JFA1 (Fig. 2c). In contrast, JFA2 activated only PYR1 and PYL1 at low concentrations (the relative activity compared with that induced by ABA were 0.47- and 0.21-fold, respectively, Fig. 2c), while JFA2 was able to activate these receptors at a concentration 3 times lower than that of JFA1. As in a previous study, Pyr exhibited a high reactivity to only PYR1 and PYL1, and quinabactin showed reactivity not only to these receptors, but also to PYL2 (Fig. 2c). These results revealed that the selectivity of JFA1 and JFA2 for the receptors was similar to Pyr, but JFA2 had a higher reactivity to the receptors than did JFA1 and Pyr.

To understand the reactivity of JFA1 and JFA2 to the ABA receptor, we performed a computational docking analysis of PYR1 and two compounds. Because the selectivity of the three compounds for the receptors were similar, we assumed that binding modes of JFA1 and JFA2 were similar to that of an ABA receptor agonist Pyr with PYR1 because of their sulfonamide. Therefore, we used 3NJO^38^ as a template for a computational docking study of PYR1 with JFA1 or JFA2. In both models, JFA1 and JFA2 were positioned on the Pyr binding pocket of PYR1 (3NJO) with similar interaction patterns that are also observed in 3NJO between two charged residues (Lys59 and Glu94) and sulfonamide. To confirm the results, we also performed additional studies using a similar but different template with a different mode (see Supplementary Methods and Table 1). The results for both docking modes suggest that stable and similar interaction patterns between Glu94 and sulfonamide of JFA1/JFA2/Qui occur at the binding pocket of PYR1, and the results of a computational docking analysis supported our biochemical analysis data.

### Functional analysis of JFAs in the ABA receptors-ABI1-SnRK1.1 pathways reconstituted in the cell-free system

**Figure 3:**
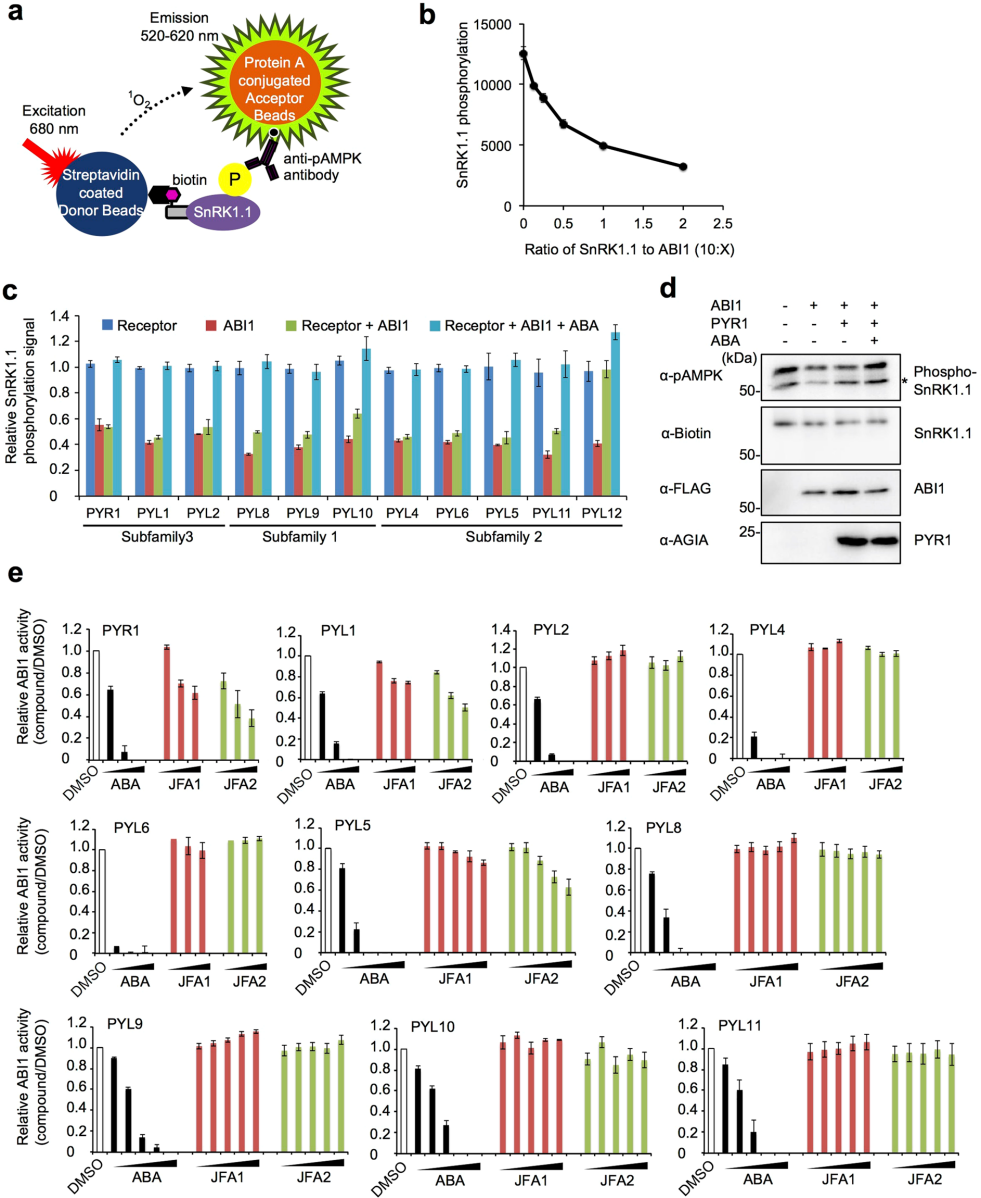
Analysis of the specificity of JFA1 and JFA2 compounds to ABA receptors based on ABA-dependent inhibition of ABI1 activity. (**a**) Principle of ABI1-dependent dephosphorylation assay of SnRK1.1 by the AlphaScreen using anti-phospho AMPK antibody. (**b**) ABI1-dependent dephosphorylation analysis of biotinylated SnRK1.1. The amount of FLAG-ABI1 was set with the volume ratios (μL) to biotinylated SnRK1.1 as 0–2:10. (**c**) Analysis of ABA receptor mediated inhibition of ABI1 activity. Biotinylated SnRK1.1 was incubated with or without C-terminal AGIA-tagged ABA receptors (PYR1, PYL1, 2, 4, 5, 6, 8, 9, 10, 11, and 12), FLAG-ABI1, or 1 μM ABA. Then, the phosphorylation level of SnRK1.1 was analyzed by using the AlphaScreen. The relative SnRK1.1 phosphorylation signal was expressed as a relative value with the signal of the mock control (SnRK1.1 only) as one. (**d**) Analysis of PYR1 mediated inhibition of ABI1 activity by immunoblot. Biotinylated SnRK1.1 was incubated with (+) or without (−) PYR1-AGIA, FLAG-ABI1, or 1 μM ABA. Phosphorylation of SnRK1.1 was analyzed by using the anti-phospho AMPK antibody. Asterisk indicates non-specific band. (**e**) Analysis of ABA receptor mediated inhibition of ABI1 activity in the presence of ABA, JFA1 or JFA2. Biotinylated SnRK1.1 was incubated with C-terminal AGIA-tagged ABA receptors (PYR1, PYL1, 2, 4, 5, 6, 8, 9, 10, and 11) and FLAG-ABI1. PYR1, PYL1, PYL4, and PYL6 were incubated with various concentrations of each compound (1, 10, or 100 μM), and PYL5, PYL8, PYL9, PYL10, and PYL11 were incubated with various concentrations of each compound (0.01, 0.1, 1, 10, or 100 μM). Relative ABI1 activity was expressed as a relative value with the signal of the DMSO (1%) control as one. Error bars represent standard deviations (*n* = 3).

Next, we analyzed the function of JFA agonists to receptors that interact with ABI1 in an ABA-independent manner. It is clear that ABI1 interacts with SnRK1s and SnRK2s and dephosphorylates phosphorylated serine (Ser)/threonine (Thr) residues on their activation loop^16,39,40^. We had already established a method to analyze the autophosphorylation of protein kinase and protein phosphatase-specific dephosphorylation with high sensitivity by using a wheat cell-free system and the AlphaScreen^26^. Therefore, we attempted to establish an assay system that could detect inhibition of ABI1 activity by ABA and ABA receptors by applying this method. SnRK1s have a structural similarity with AMP activated protein kinase (AMPK) in animals. A previous study showed that phosphorylation and ABI1-dependent dephosphorylation of Thr175 on the activation loop of SnRK1.1 could be detected with an anti-phospho-AMPKα (Thr172) antibody^40^. Thus, we analyzed ABI1-dependent dephosphorylation of SnRK1.1 by using the phosphorylation analysis method based on a wheat cell-free system using an anti-phospho-AMPKα antibody (Fig. 3a). A Thr175 of mono-biotinylated SnRK1.1 was phosphorylated by endogenous protein kinase in a wheat germ extract, and the AlphaScreen signal of SnRK1.1 phosphorylation was decreased by the additional ABI1 in a dose-dependent manner (Fig. 3b), indicating that ABI1-dependent dephosphorylation could be detected by the AlphaScreen. To investigate whether ABA receptor and ABA-dependent inhibition of ABI1 activity could be detected by this assay, we selected 11 receptors, including five receptors activated by JFA1 and/or JFA2 and six receptors that interacted with ABI1 in an ABA-independent manner. The receptors (PYR1, PYL1, PYL2, PYL4, and PYL6) that required ABA for interaction with PP2CAs showed inhibition of ABI1 activity only in the presence of ABA (Fig. 3c). Furthermore, ABA-dependent inhibition of ABI1 activity by PYR1 was also confirmed by immunoblot analysis (Fig. 3d), indicating that this assay could detect ABA and ABA receptor-dependent ABI1 inhibition. Conversely, the receptors (PYL5 and PYL8-11) that do not require ABA for interaction with PP2CA slightly inhibited ABI1 activity in the absence of ABA, and their activities were completely inhibited in the presence of ABA. Previous studies have shown that these receptors could interact and slightly inhibit ABI1 in the absence of ABA, and that ABA enhanced inactivation of ABI1^24,34^. Thus, these previous findings support our data. However, PYL12 showed dramatic inhibition of ABI1 activity in the absence of ABA (Fig. 3c), suggesting that PYL12 inhibits the ABI1-mediated ABA signal in an ABA-independent manner.

Next, we evaluated the functions of JFA1 and JFA2 relative to the ABA receptors. Phosphatase inhibition assays revealed that JFA1 induced only PYR1 and PYL1-mediated inhibition of ABI1 activity, and JFA2 induced PYR1, PYL1, and PYL5-mediated inhibition of ABI1 activity (Fig. 3e). In contrast, JFA1 and JFA2 exhibited no effect on other receptors (Fig. 3e). In combination, ABA receptor-ABI1 interaction analyses (Fig. 2) and the phosphatase inhibition analysis (Fig. 3e) revealed that JFA1 was a selective agonist for PYR1 and PYL1, while JFA2 was a selective agonist for PYR1, PYL1, and PYL5. Furthermore, JFA2 had more reactivity to PYR1 and PYL1 than JFA1.

### Comparison analysis of gene expression profiling between ABA and JFA2 treatments

**Figure 4:**
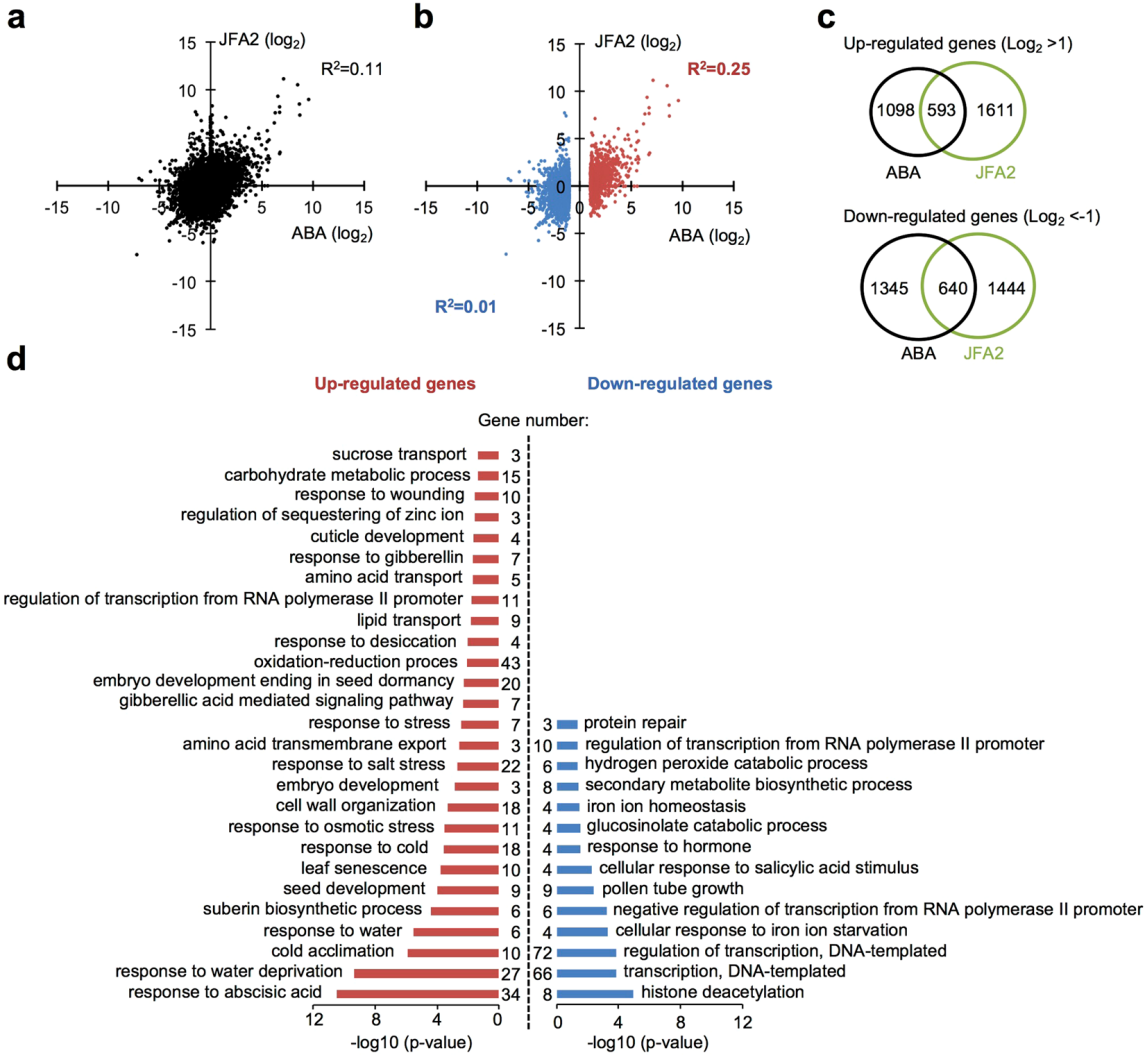
Genome-wide comparison of ABA and JFA2 in *Arabidopsis* plants by RNA-sequencing. (**a,b**) Comparison of expression levels induced by ABA and JFA2 treatments. The axes plot log_2_-transformed values of differential expression gene responses to ABA or JFA2 relative to the DMSO control treatment. All expressed genes were indicated as dots on the black background (**a**). Red dots or blue dots represent differentially expressed genes (log_2_ fold change >1 or <−1) in response to ABA (**b**). The coefficient of determination (*R*^2^) between ABA and JFA2 was calculated and indicated in the figure with corresponding colors. (**c**) Venn diagrams showing the overlap of the upregulated (log_2_ fold change >1) and downregulated (log_2_ fold change <−1) genes between ABA and JFA2 treatments. (**d**) Gene ontology analysis of differentially expressed genes. Gene ontological analysis of 1,232 differentially expressed genes (log_2_ fold change >1 or <−1) responsive to both ABA and JFA2 treatments was performed using DAVID with EASE score (*p*-value ≤ 0.05). Each biological process is listed based on its *p*-value.

To compare the biological activity of JFAs, gene expression profiles were performed by RNA-sequencing using JFA2 or ABA as references. Twenty-day-old *Arabidopsis* plants were treated with 50 μM of JFA2 or ABA. Transcriptome analysis revealed a slight correlation between ABA and JFA2 (*R*^2^ = 0.11, Fig. 4a). However, correlation with JFA2 was revealed in genes whose expression levels increased by more than 2-fold after ABA treatment (*R*^2^ = 0.25, Fig. 4b), but it was not observed in genes downregulated by ABA treatment (*R*^2^ = 0.01, Fig. 4b). Compared with the DMSO-treatment as the control sample, 1,691 and 2,204 genes were induced more than 2-fold in ABA or JFA2-treatment, respectively (Fig. 4c). Among them, 593 genes were induced more than 2-fold in both ABA and JFA2. Conversely, 1,985 and 2,084 genes were suppressed more than 2-fold in ABA or JFA2-treatment, respectively, of which 640 genes were common in ABA and JFA2-treatments (Fig. 4c). Gene ontology analysis of both ABA and JFA2 upregulated and downregulated genes revealed that, similar to ABA, JFA2 mainly induced ABA responses, dehydration, and cold-stress related genes (Fig. 4d).

### Biological functional analysis of JFA1 and JFA2 in *Arabidopsis* seeds and plants

**Figure 5:**
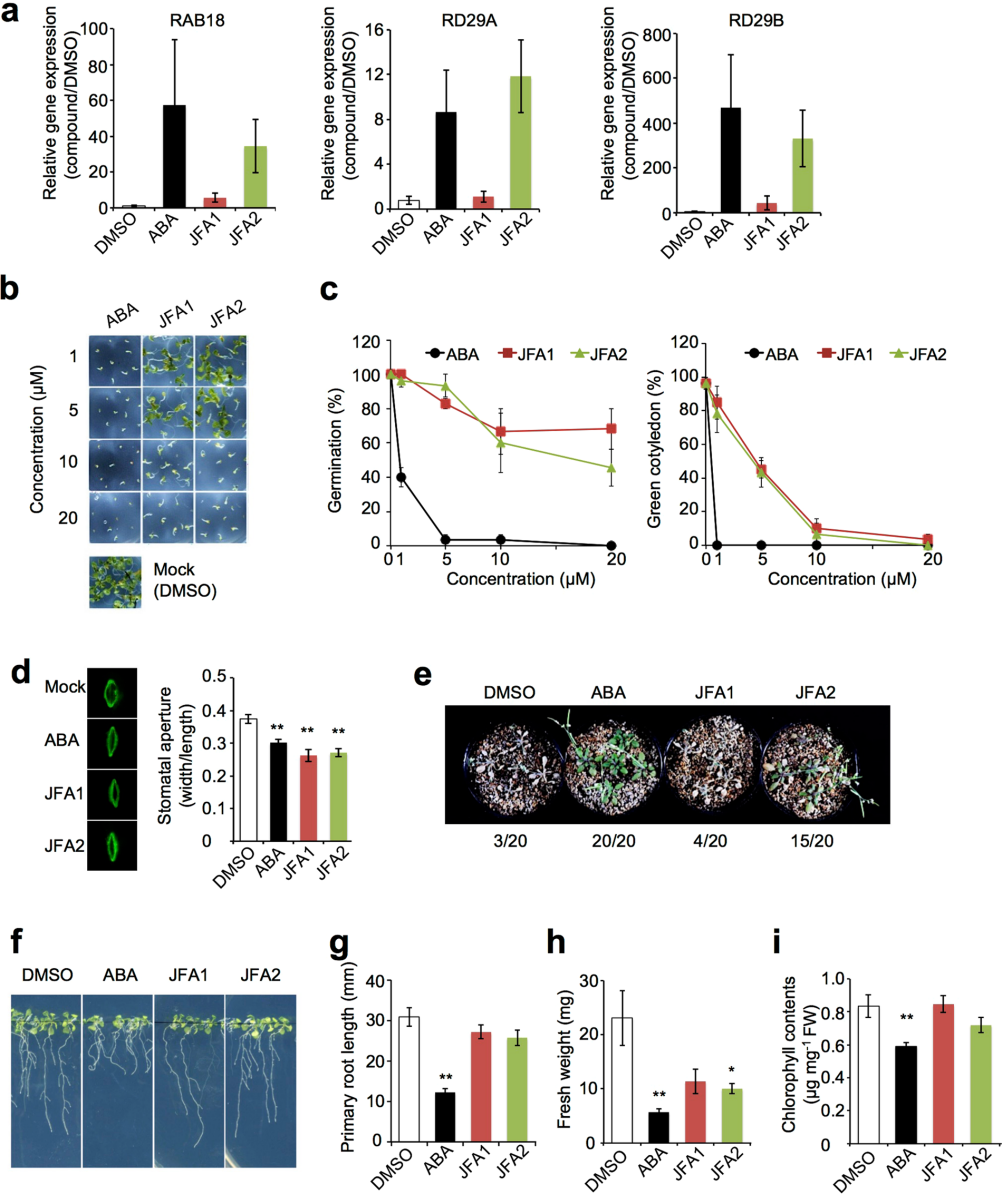
Biological functional analysis of JFAs in *Arabidopsis*. (**a**) Expression of *RAB18*, *RD29A*, and *RD29B* genes after treatment with ABA, JFA1, or JFA2. Twenty-day-old *Arabidopsis* plants were treated with 50 μM ABA, JFA1, or JFA2 for 5 h. Transcript levels were analyzed by RT-qPCR and normalized to the level of ACTIN4, and the relative transcript level in the DMSO treated sample was one. Error bars represent standard deviation (*n* = 3). (**b**,**c**) Effects of ABA and JFA1/2 on seed germination efficiency. Seeds were germinated on MS medium supplemented with the indicated concentrations of ABA, JFA1, or JFA2 for 8 d (**b**). The seed germination and green cotyledon expansion rates were determined by counting (**c**). Germination/green cotyledon expansion rates were calculated from three independent experiments with 10 seeds per treatment. Error bars represent standard deviation. (**d**) Stomatal closure test. Leaves of 4-week-old *Arabidopsis* plants were incubated in a stomatal opening solution for 2 h followed by treatment with 25 μM ABA, JFA1, or JFA2 for 1 h. Stomata were visualized by Rhodamine 6G stain (left). Stomatal aperture measurement was carried out by recording the width to length ratio (right). Stomatal aperture indexes were calculated from three independent experiments with 30 stomata per treatment. Error bars represent standard errors. (**e**) Effect of ABA and JFA1/2 on *Arabidopsis* drought tolerance. *Arabidopsis* plants were grown for 2 weeks before water withholding. Then plants were treated every 3 d with 25 µM ABA, JFA1, or JFA2. After 2 weeks of drought treatment, plants were rehydrated. The number of surviving plants was determined 2 d after re-watering. (**f**–**i**) Effects of ABA and JFA1/2 on *Arabidopsis* seedling growth. Seven-day-old seedlings were transferred and grown on MS medium containing with 25 μM ABA, JFA2, or JFA2 for 10 d (**f**). The primary root lengths (**g**), fresh weights (**h**), and chlorophyll contents (**i**) were measured. The measured values were based on three independent experiments with 5 seedlings per treatment. Error bars represent standard errors. Statistically significant changes, compared with the mock control (DMSO), are indicated (*P < 0.05, **P < 0.01, two-tailed Student’s t-test) (**a**,**b**,**g**–**i**).

Transcriptome analysis revealed that JFA2 could induce some genes under the control of ABA signaling (Fig. 4). Next, we confirmed whether ABA-induced genes were induced by JFA2 and JFA1 in *Arabidopsis* plants. RT-qPCR analysis revealed that the well-characterized ABA-induced genes, *RAB18*^41^, *RD29A*^42^, and *RD29B*^43^, were dramatically induced by JFA2 treatment but were slightly induced by JFA1 (Fig. 5a). ABA is known to be involved in inhibition of seed germination, growth inhibition, and stress responses, such as response to drought^1^. Thus, we investigated the function of JFA1/JFA2 in these phenotypes associated with ABA response. *Arabidopsis* seed germination and cotyledon greening of seedlings were completely inhibited at 1 μM concentration of ABA (Fig. 5b,c). In contrast, both JFA1 and JFA2 suppressed approximately half of the seed germination at 20 μM, while cotyledon greening of seedlings was completely inhibited at the same concentration (Fig. 5b,c). In addition to seed germination, treatment of JFA1 and JFA2 induced stomatal closure in detached and intact *Arabidopsis* leaves (Fig. 5d, Supplementary Fig. 5). Furthermore, long-term treatment with JFA2, but not with JFA1, showed increased drought tolerance compared to the control treatment (Fig. 5e). The seedling growth assay revealed that ABA dramatically inhibited primary root growth. However, both JFA1 and JFA2 did not inhibit primary root growth (Fig. 5f,g). Growth of the aboveground parts was slightly suppressed by JFA1 and JFA2 treatments (Fig. 5h), but the ABA-dependent reduction in chlorophyll concentration was not observed (Fig. 5i). These results suggested that JFA2 has the ability to function like ABA in inducing signaling in seeds and plants, but JFA2 does not inhibit growth compared to ABA. In contrast, JFA1 may activate ABA signals in seeds and stomata, but does not have sufficient capacity to adapt to long-term drought stress. These results indicate that JFA2 is a new ABA receptor agonist without inhibitory effects of root growth.

## Discussion

In this study, we developed a highly sensitive, specific, and high-throughput analysis method for biochemical functions of ABA receptors using a wheat cell-free system combined with the AlphaScreen. Although several studies have reported functional analysis assay methods for ABA receptors using the AlphaScreen^21,44–46^, our assay system has several advantages. In many cases, proteins are synthesized by *Escherichia coli* and need to be purified for analysis. It is difficult to purify multiple proteins in a functional state and to simultaneously analyze their biochemical functions. However, the wheat cell-free system used in this study does not require protein purification for analysis, and thus it is possible to analyze the functions of multiple proteins at the same time. In addition, previous studies using the AlphaScreen used approximately 100 nM purified proteins for the assay^21,44–46^, but our assay system was able to analyze unpurified crude solutions containing 20 nM proteins. Therefore, our assay system is useful for screening chemical libraries for compounds.

Using our assay method, we uncovered the biochemical ABA interactome among ABA receptors-PP2CAs and ABA receptor-ABA receptor with or without ABA. The results of the interaction analysis between the 14 receptors and 7 PP2CAs exhibited 83.1% commonality with previous proteome data and revealed nine new interactions. Recently, a study using a cell reporter assay based on *Arabidopsis* protoplast transient expression analysis showed that 113 pairs of ABA receptors-PP2CAs are functional in the ABA signal^23^. This cell-based reporter assay data probably complements and supports our *in vitro* interaction analysis data. Using homo/hetero dimerization analysis of ABA receptors, a total of 24 interaction pairs, including 7 new homodimer interactions and 13 heterodimer interactions, were detected. The interaction signals of most of these pairs were decreased by ABA treatment; however, interactions of PYL5-PYL10 and PYL9-PYL11 were enhanced by ABA treatment. These results suggested that the majority of the homo/hetero-dimerization of the ABA receptor functions as a suppressive mechanism of the ABA signal at low ABA levels, but specific ABA receptors, PYL5 and PYL9-11, possess the ability to prevent extreme ABA signaling by dimerization under high ABA level conditions. However, to confirm their biochemical results, we need to investigate whether the interactions observed here can also be confirmed in truly intact plants. Although our assay system is probably effective for analysis of ABA receptors-PP2CAs and ABA receptors-ABA receptors interaction, some of the networks revealed by the proteome data and the cell-based reporter assay^23^ data were not confirmed by this assay. Indeed, this may have been undetectable in the present study due to low protein expression levels and/or unknown modification(s) of proteins in the cells and plants.

By applying the biochemical analysis method of the ABA receptor for chemical screening, we constructed the wheat cell-free-based drug screening system that can screen agonist compounds for ABA receptors with high sensitivity. Our cell-free based screening system, using the translational mixtures (1 µL) from the wheat cell-free system without protein purification, exhibited high quality (Z′-factor = 0.88 ± 0.02) and high-throughput performance (9,600 compounds/3 h). Indeed, we identified two ABA receptor agonists JFA1 and JFA2. The ABA receptor-PP2CAs interaction assay and phosphatase inhibition assay revealed that JFA1 has the ability to activate PYR1 and PYL1, and that JFA2 has the ability to activate PYR1, PYL1, and PYL5. Furthermore, it was revealed that JFA2 has more reactivity to PYR1 and PYL1 than JFA1. Similar to several known ABA receptor agonists, such as pyrabactin and quinabactin, JFA1 and JFA2 have sulfonamide as a common structure that probably interacts with the pocket of the ABA receptor. In contrast, PYL4, 6 and 11 showed JFA1/2-dependent binding with ABI1, but could not inhibit ABI1 activity in the presence of JFA1/2. Previous research revealed that pyrabactin weakly induces the interaction between PYL3-HAB1 but hardly inhibits the HAB1 activity^47^. Furthermore, the crystal structure analysis showed that pyrabactin induces the closure of the gate part of PYL3, but its degree of closure is excessive compared with that induced by ABA, and does not lead to complex formation, which leads to efficient inhibition of HAB1 activity. These results suggested that proper closure of the gate part of the receptor is necessary for ABA receptor-PP2CA complex formation and inhibition of the PP2CA activity. Based on these findings, although JFA1/2 induced the interaction between PYL4, 6, 11 and ABI1, it may lead to the formation of an inappropriate complex, which could not inhibit ABI1 activity. JFA2-treatment induced the expression of ABA responsive genes in *Arabidopsis* plants, but JFA1 barely induced them. However, both JFA1 and JFA2 inhibited seed germination and cotyledon greening of the seedlings. These results suggested that PYR1 and PYL1 are mainly involved in ABA signaling in the seeds and seedlings, but are not predominant in the ABA signals that cause ABA-induced gene expression in plants. Pyrabactin, a selective agonist for PYR1 and PYL1, also only inhibited seed germination and seedling greening^24^. Interestingly, not only JFA2 but also JFA1 induced stomatal closure; however, only JFA2 promoted drought tolerance. This phenomenon was consistent with the results of the intact-plant assay that showed that JFA2 strongly induced stomatal closure compared with JFA1 (Supplementary Fig. 5). A glucuronidase reporter assay-based promoter activity analysis showed that PYR1, PYL1, and PYL5 were expressed in guard cells^48^. These results suggested that it is necessary to induce both the stomatal closure and the expression of genes involved in stress tolerance to adapt to the long-term drought stress, and that activation of PYL5 is important for drought stress tolerance. Recent studies have shown that the selective agonist 6-nor-ABA for PYL5 and PYL6 promoted drought tolerance^49^, while PYL5 over-expression led to enhanced ABA-induced stomatal closure and drought tolerance in *Arabidopsis* plants^50^. Conversely, JFA1 and JFA2 slightly inhibited the growth of above-ground parts, but did not inhibit root growth. These results suggested that PYR1, PYL1, and PYL5-dependent ABA signal are involved in growth inhibition in the aboveground parts, but are not involved in root growth inhibition. Thus, JFA1 and JFA2 inhibit seed germination and cotyledon greening of seedlings by activating PYR1 and PYL1, and JFA2 is able to enhance drought tolerance without inhibiting root growth by activating not only PYR1 and PYL1 but also PYL5. Nevertheless, it is not clear whether activation of PYL5 alone or multiple ABA receptors is necessary for drought tolerance; however, this may be clarified by analyzing the *pyl*5 mutant or designing a PYL5 selective agonist. Development of selective agonist compounds could be expected to lead to the elucidation of the complex signal networks of ABA receptors and the development of new agrochemicals aimed at improving stress tolerance and adaptation. We are convinced that our assay system is one of the useful approaches to such studies.

## Materials and Methods

### Plant material

The *Arabidopsis thaliana* ecotype Columbia (Col-0) was used in this study. Dry seeds were stored under dark conditions at 4 °C for 1 year. The plants were grown in a growth cabinet under long days (16-h light/8-h dark) at 22 °C.

### Chemicals

ABA (Tokyo Chemical Industry), JFA1 (Life Chemicals), JFA2 (developed in this study), QFA (also developed in this study), pyrabactin (Sigma), and quinabactin (Life Chemicals) were prepared as stock solutions of 100 mM in DMSO, and appropriately diluted just before use. The final concentration of DMSO in the culture medium or assay buffer was 1% or less.

### Synthesis of JFA2 and QFA

For details, see Supplementary Methods.

### Construction of the *in vitro* transcription templates

For details, see Supplementary Methods.

### Wheat cell-free protein synthesis

*In vitro* transcriptions and translations were performed by using the bilayer method using the WEPRO1240 expression kit (Cell-Free Sciences) according to the manufacturer’s instructions. Biotin labeling was carried out by using a method described previously^51^. Specifically, at the time of the translation reaction, biotin ligase (BirA) synthesized by a wheat cell-free system was added to the bottom layer and incubated in the presence of 0.5 μM of d-biotin (Nacalai Tesque). The aliquots were used for the expression analysis and functional characterization.

### Interaction analysis of ABA receptors-PP2CAs or ABA receptors

For the AlphaScreen-based protein–protein interaction analysis of biotinylated ABA receptors and FLAG-tagged PP2CAs or AGIA-tagged ABA receptors in the presence of ABA or ABA receptor agonists, we synthesized N-terminal mono-biotinylated or AGIA-tagged^29^ ABA receptors (PYR1 and PYL1-13) and N-terminal FLAG-tagged PP2CAs (ABI1, ABI2, HAB1, HAI1, HAI2, AHG1, and AHG3) by using a wheat cell-free system. The AlphaScreen-based protein–protein interaction analysis was performed by a slightly modified method described previously^27^. Fifteen microliters of a reaction mixture containing AlphaScreen buffer (100 mM Tris-HCl (pH 8.0), 0.1% Tween20, and 1 mg/mL bovine serum albumin (BSA)), 0.5 μL of mono-biotinylated ABA receptor, 0.5 μL of FLAG-tagged PP2CA or AGIA-tagged ABA receptor, and various concentrations (indicated in figure legends) of ABA, JFA1, JFA2, QFA, pyrabactin, or quinabactin was added to a 384-well Optiplate (PerkinElmer). After incubation at 25 °C for 1 h, 10 μL of a detection mixture containing AlphaScreen buffer, 0.1 μL of streptavidin-coated donor beads, 0.1 μL of protein A-coated acceptor beads, and 5 μg/mL anti-FLAG M2 antibody (Sigma-Aldrich) was added to each well. Thereafter, the plate was incubated for an additional 1 h. Luminescence signals were detected by using the Envision plate reader (PerkinElmer). The experiment was repeated three times, and the data are presented as average values. Control samples were dihydrofolate reductase (DHFR) of *E. coli* or a mock treatment (1% DMSO). We defined an interaction pair as that occurring when its signal was at least five times (for interaction analysis of ABA receptors-PP2CAs) or two times (for interaction analysis of ABA receptors-ABA receptors) higher than that of the DHFR.

### Chemical library screening

For details, see Supplementary Methods.

### AlphaScreen-based protein phosphatase assay

For the AlphaScreen-based protein phosphatase activity analysis of ABI1, we synthesized the N-terminal mono-biotinylated SnRK1.1^26^, N-terminal AGIA-tagged ABA receptors (PYR1, PYL1, 2, 4-6, and 8-12), and FLAG-ABI1 by using the cell-free system. Phosphatase activity analysis was performed by slightly modifying a method described previously^26^. Ten microliters of phosphatase reaction mixture containing the AlphaScreen buffer, 1 mM MnCl_2_, and 1 μL of biotinylated SnRK1.1 in the presence or absence of 1 μL of AGIA-tagged ABA receptor, and 0.1 μL of FLAG-ABI1, and various concentrations (indicated in figure legends) of ABA or ABA receptor agonists (with a final DMSO concentration of 1%) were added to a 384-well Optiplate. After incubation at 25 °C for 1 h, 15 μL of a detection mixture containing AlphaScreen buffer, 83 mM NaCl, 3.3% Brij 35, 0.1 μL of streptavidin-coated donor beads, 0.1 μL of protein A-coated acceptor beads, and 5 μg/mL anti-phospho-AMPKα (Thr172) (clone 40H9) antibody (Cell signaling) was added to each well. Thereafter, plate was incubated for an additional 1 h. Luminescence signals were detected by using the Envision plate reader (PerkinElmer). The experiment was repeated three times. The data represent average values.

### Docking study of *Arabidopsis* ABA receptor PYR1 with JFA/JFA2

For details, see Supplementary Methods.

### Transcriptome sequencing (RNA-seq) analysis and gene ontology biological process enrichment analysis

For details, see Supplementary Methods.

### Gene expression analysis by reverse transcription quantitative PCR

Reverse transcription quantitative PCR (RT-qPCR) was performed by using a slightly modified method described previously^27^. Twenty-day-old plants, after germination on half-strength MS agar plates, were sprayed with chemical solutions containing 50 μM ABA, JFA1, or JFA2 and 0.04% Silwet L-77. After incubation for 5 h, total RNA was extracted from above-ground parts of plants using TRI Reagent (Sigma). First-strand cDNA synthesis and RT-qPCR were performed using KOD SYBR qPCR/RT Set III (TOYOBO) according to the manufacturer’s instructions. Amplified products were detected by using a real-time LightCycler96 PCR system (Roche). Relative gene expression levels were normalized to the ACTIN4 (at5g59370) as an internal control gene. Gene-specific primers are listed in Supplementary Table 2.

### Growth assay

For the growth assay, seeds were surface sterilized with commercial bleach and washed three times with sterile water. The seeds were sown on germination medium agar plates (without sucrose) and kept at 4 °C for 3 days, and then incubated in a growth cabinet for 7 days. Seedlings were transferred on half-strength Murashige and Skoog (MS) agar plates (1% sucrose) supplemented with 25 μM ABA, JFA1, or JFA2. The plants were grown in a vertical position under standard conditions for 10 days, and then pictures of each plate were taken. Primary root length, plant weight, and chlorophyll concentration^52^ were measured.

### Germination assay

For the germination assay, seeds surface-sterilized by the same method as above were sown on half-strength MS agar plates (2% sucrose) supplemented with ABA, JFA1, or JFA2. After being stratified at 4 °C for 3 days, the plates were transferred to a growth cabinet and incubated for 8 days. Photographs were also taken, and the germination rates (defined by radicle protrusion) and green cotyledon expansion rates were determined 8 days after stratification.

### Drought stress assay

The seeds were imbibed under dark conditions at 4 °C for 2 days and planted directly in the soil. Plants were grown in a growth cabinet under normal watering conditions. After 2 weeks, the drought-stress treatment was initiated by withholding water for 14 days. During the drought-stress treatment, plants were sprayed with chemical solutions containing 25 μM ABA, JFA1, or JFA2 and 0.02% Silwet L-77 every 3 days. The plants were watered after 14 days of drought stress, and the surviving plants were counted 2 days later.

### Stomatal aperture analysis

Images of stomatal apertures were obtained by using the rhodamine 6G-staining method^53^. For analysis of stomatal aperture, leaves were detached from rosette leaves of 4-week-old plants and incubated in the opening buffer (5 mM KCl, 10 mM MES (2-(*N*-morpholino)ethanesulfonic acid), 50 μM CaCl_2_, pH 6.15) in Petri dishes for 2 h to open the stomata. Then, the leaves were treated with the opening buffer containing 25 μM ABA, JFA1, or JFA2 for 2 h. Subsequently, the leaves were treated with the opening buffer containing with 1 μM rhodamine 6 G (Sigma) for 2 min. For intact-plant assay, 4-week-old plants were sprayed with chemical solutions containing 25 μM ABA, JFA1, or JFA2 and 0.02% Silwet L-77. Then, the plants were incubated in a growth cabinet at 22 °C under light conditions. After incubation for 3, 6, and 24 h, leaves were detached and treated with water containing 1 μM rhodamine 6 G (Sigma) for 2 min. Imaging of stomata was performed with a fluorescence microscope IX-73 (Olympus). Image analysis was performed using the ImageJ software (https://imagej.nih.gov/ij/). The width and the length of the stomatal aperture were measured, and the stomatal aperture index was calculated by dividing the aperture the length by the width. The stomatal aperture index of at least 10 stomata per leaf was calculated, with three leaves per treatment used for statistical analysis.

### Statistical analysis

All experiments were repeated at least three times unless otherwise specified. Sample size for each experiment is indicated in the figure legends. Statistical significance was calculated using two-sided, unpaired Student’s t-tests in Microsoft Excel spreadsheets with basic statistical analysis program. The coefficient of determination (*R*^2^) between two data was calculated using Excel spreadsheet. All uncropped blot images are provided in Supplementary Fig. 6.

## Electronic supplementary material

Supplementary information
