# Supplementary material for: Identification of new abscisic acid receptor agonists using a wheat cell-free based drug screening system
Source: Sci Rep. 2018 Mar 9;8:4268. doi: 10.1038/s41598-018-22538-9 (PMC5844987; doi:10.1038/s41598-018-22538-9)
Supplement: Supplementary file 1 — Supplementary information [file 41598_2018_22538_MOESM1_ESM.pdf]

Identification of new abscisic acid receptor agonists using a wheat cell-free based drug screening system

Keiichirou Nemoto[1], Makiko Kagawa[1], Akira Nozawa[1], Yoshinori Hasegawa[2], Minoru Hayashi[3], Kenichiro Imai[4], Kentaro Tomii[4] and Tatsuya Sawasaki[1]*

[1] Proteo-Science Center, Ehime University, 3 Bunkyo-cho, Matsuyama, Ehime 790-8577, Japan, [2] Department of Technology Development, Kazusa DNA Research Institute, Kisarazu, Chiba 292-0818, Japan, [3] Department of Materials Science and Biotechnology, Graduate School of Science and Engineering, Ehime University, 3 Bunkyo-cho, Matsuyama 790-8577, Japan, [4] Artificial Intelligence Research Center (AIRC) and Biotechnology Research Institute for Drug Discovery, National Institute of Advanced Industrial Science and Technology (AIST), 2-4-7 Aomi, Koto Ward, Tokyo 135-0064, Japan.

*Corresponding Author:

Tatsuya Sawasaki

E-mail: sawasaki@ehime-u.ac.jp.

Supplementary Methods

Synthesis of JFA2 and QFA

For synthesis of *N*-(1,2,3,4-tetrahydro-2-oxo-6-quinolinyl)-4-trifluoromethoxybenzenesulfonamide (QFA, quinoline and fluorine containing ABA receptor activator), 6-amino-3,4-dihydro-2(1H)-quinolinone, (20.5 mg, 0.126 mmol) was dissolved in dichloromethane (0.6 mL), and triethylamine (42.4 mg, 0.419 mmol) was added while stirring followed by dropwise addition of 4-trifluoromethoxybenzenesulfonyl chloride, (35.9 mg, 0.138 mmol). After stirring at room temperature overnight, water was added to the reaction mixture. The mixture was washed successively with water, 1N HCl aq. (twice), saturated sodium hydrogen carbonate solution and brine. The organic layer was dried over anhydrous sodium sulfate and evaporated to dryness in vacuo. The crude product was dissolved in ethyl acetate and subjected to silica-gel column chromatography (hexane-ethyl acetate as the eluent) to afford the product as a colorless solid (18.2 mg, 37 %). ^{1}H NMR (400 MHz, $CDCl_3$) δ 7.85 – 7.77 (m, 2H), 7.33 – 7.25 (m, 2H), 6.73 (s, 1H), 6.70 (s, 1H), 6.63 (s, 1H), 3.88 – 3.80 (t, *J* = 6.2 Hz, 2H), 2.79 (dd, *J* = 8.8, 6.1 Hz, 2H), 2.69 (t, *J* = 6.2 Hz, 2H), 2.61 (dd, *J* = 8.8, 6.1 Hz, 2H), 1.90 (quint, *J* = 6.2 Hz, 2H).

For synthesis of N-(2,3,6,7-tetrahydro-3-oxo-1H,5H-benzo[ij]quinolizin-9-yl)-4-trifluoromethoxybenzenes

ulfonamide (JFA2, julolidine and fluorine containing ABA receptor activator 1), 9-amino-2,3,6,7-tetrahydro-1*H*,5*H*-benzo[*ij*]quinolizine-5-one (5.2 mg, 0.026 mmol) was dissolved in dichloromethane (0.1 mL), and triethylamine (3.8 mg, 0.038 mmol) was added while stirring followed by dropwise addition of 4-trifluoromethoxybenzenesulfonyl chloride (8.8 mg, 0.034 mmol). After stirring at room temperature for 10 h, water was added to the reaction mixture. The mixture was washed successively with water, 1N HCl aq. (twice), saturated sodium hydrogen carbonate solution, and brine. The organic layer was dried over anhydrous sodium sulfate and evaporated to dryness in vacuo. The crude product was dissolved in ethyl acetate and subjected to preparative TLC on silica-gel (hexane-ethyl acetate as the eluent) to afford the product as colorless solid (5.7 mg, 50 %). ^{1}H NMR (400 MHz, $CDCl_3$) δ 8.17 (s, 1H), 7.83 – 7.74 (m, 2H), 7.31-7,26 (m, 2H), 6.97 (s, 1H), 6.94 (br s, 1H), 6.83 (dd, J = 8.4, 2.4 Hz, 1H), 6.65 (d, J = 8.4 Hz, 1H), 2.91 (t, J = 7.6 Hz, 2H), 2.61 (t, J = 7.6 Hz, 2H).

Construction of the *in vitro* transcription templates

The genes of PYR1, PYL1, PYL2, PYL4-6, PYL8, and PYL11 were amplified by PCR from the RIKEN *Arabidopsis* full-length (RAFL) cDNA library. The genes of PYL3, PYL7, PYL9, PYL10, PYL12, and PYL13 were amplified by PCR using total cDNA prepared from mRNA of Col-0 plant as a template (described below).

The DNA fragments of the open-reading frame (ORF) were cloned in a pDONR221 vector using the gateway cloning system (Thermo Fisher Scientific). After confirming sequences, we generated these expression vectors by LR Clonase recombination with pEU-His-bls-GW vectors for *in vitro* transcription. Then, these regions of the gene containing ORF and tag sequence were amplified by PCR and used as transcription templates.

For preparation of *in vitro* transcription templates for FLAG-PP2CAs and biotinylated SnRK1.1, the genes of ABI1, ABI2, HAB1, HAI1, HAI2, AHG1, AHG3, and SnRK1.1 were amplified by the split-primer PCR method[1] using the RAFL cDNA library as the template. For preparation of *in vitro* transcription templates for AGIA-tagged ABA receptors, gene regions of ABA receptors were amplified by the split-primer PCR method[1] using the genes sub-cloned into pDONR 221 as templates. All primer sequences and plasmids used in this study are listed in supplementary Table 2 and Table 3.

Chemical library screening

For the AlphaScreen-based chemical library screening, we used a diverse set of 9,600 synthesized chemicals established by the Drug Discovery Initiative (The University of Tokyo, Tokyo, Japan). All compounds dissolved in DMSO were predispensed on a 384-well Optiplate (250 nL/well) with the concentration of 100 μM,

and then 15 μL of PYR1/ABI1 mixture containing AlphaScreen buffer, 1 μL of biotinylated PYR1, 1 μL of FLAG-ABI1, and 1 μM ABA (0.067% DMSO), or 0.067% DMSO were dispensed to each well by using a FlexDrop dispenser (PerkinElmer). These reaction mixtures contained DMSO at a 1.73 % final concentration. Each 384-well plate contained 16 negative control wells (containing biotinylated PYR1 and FLAG-DHFR) and 16 positive control wells (containing biotinylated PYR1, FLAG-ABI1, and 1 μM ABA). After incubation at 25 °C for 1 h, 10 μL of detection mixture were added to each well of the 384-well Optiplate by using a FlexDrop dispenser (PerkinElmer), followed by incubation at 25 °C for 1 h. The composition of the detection mixture was described above (see interaction analysis of ABA receptors and PP2CAs). The final concentration of each compound was 1 μM, ABA was 0.6 μM, and DMSO was 1% in the final solution. Luminescence was analyzed using the AlphaScreen detection program. Data were expressed as relative values normalized by the value of the AlphaScreen signal between biotinylated PYR1 and FLAG-ABI1 in the presence of ABA only.

Docking study of *Arabidopsis* ABA receptor, PYR1 with JFA1/JFA2/Qui

We used 3NJO[2] as a template for the computational docking study of PYR1 with JFA1/JFA2 because we assumed that binding modes of JFA1/JFA2 were similar to that of an ABA agonist, pyrabactin with PYR1 regarding their sulfonamide. Additionally,

we used a homology model of PYR1-quinabactin complex constructed based on PYL2-quinabactin complex (4LA3[3]) as JFA1/JFA2 is structurally similar to quinabactin than pyrabactin. Docking studies and homology modeling were performed by using Molecular Operating Environment (MOE) software package (MOE, 2014.09; Chemical Computing Group Inc., Montreal, QC, Canada) with the Amber10:EHT force field. During docking simulations, we used template forcing mode, targeting sulfonamide of pyrabactin or quinabactin, with the induced fit method, including water molecules. To confirm the results, we performed two types of induced fit modes, tether and free modes: side chains of receptor pocket are tethered with a constant weight and free to move, respectively. We chose the best models based on the GBVI/WSA dG score[4], which estimates the free energy of binding for the ligands, and summarized the results in supplementary Table 1.

Transcriptome sequencing (RNA-seq) analysis and gene ontology biological process enrichment analysis

For RNA-seq analysis, 20 day-old-plants, after germination on half-strength MS agar plate, were sprayed with chemical solutions containing 50 μM ABA, JFA1, or JFA2, and 0.04% Silwet L-77. After incubation for 5 h, above-ground part of plants were homogenized, and total RNA was extracted using TRI Reagent (Sigma). Total RNA was treated with DNase I (TaKaRa) for 30 min at 37 °C, and then purified using

the RNeasy mini kit (Qiagen). The concentration and quality of the RNA was verified with the Quantus Fluorometer (Promega) and Agilent 2100 Bioanalyzer, respectively. All the samples had RIN values over 7. Sequencing libraries were prepared according to the manufacturer's instructions for the Agilent SureSelect Strand-Specific RNA Library Prep for Illumina. Briefly, poly-A RNA was purified from 300 ng total RNA per sample using oligo dT magnetic beads. The libraries were PCR amplified for 13 cycles and purified with AMPure XP beads. Sequencing of the libraries was conducted on the Illumina HiSeq1500 system performing single-end 50 bp reads.

Each read was assessed for quality using the FASTX Toolkit (v0.0.13) software. Base trimming was done from the 3′ end of each read to remove bases with quality below Q30 up to a minimum of 25 bp. Reads shorter than 25 bp were removed before further analysis. The reads were mapped with the TAIR10 genome using Strand NGS software (v2.6, Agilent Technologies). After DeSeq normalization, we analyzed the gene expression profile across the samples.

The gene ontology biological process enrichment analysis was performed by the DAVID functional annotation clustering tool (http://david.abcc.ncifcrf.gov/home.jsp)[5]. Differentially expressed genes (1232 genes, $\log_2$ fold change > 1 or < -1) responsive to both ABA and JFA2 treatments were annotated by the *Arabidopsis* Genome Initiative (AGI) locus codes, and were analyzed for gene ontology terms (counts ≥ 2 and a modified Fisher Exact p value, EASE score $\leq$

0.05).

Supplementary References

1. Sawasaki, T., Ogasawara, T., Morishita, R. & Endo Y. A cell-free protein synthesis system for high-throughput proteomics. *Proc. Natl. Acad. Sci. USA.* **99**, 14652-14657 (2002).
2. Peterson, F.C. *et al.* Structural basis for selective activation of ABA receptors. *Nat. Struct. Mol. Biol*. **17**, 1109-1113 (2010).
3. Okamoto, M. *et al*. Activation of dimeric ABA receptors elicits guard cell closure, ABA-regulated gene expression, and drought tolerance. *Proc. Natl. Acad. Sci. USA*. **110**, 12132-12137 (2013).
4. Corbeil, C. R., Williams, C. I. & Labute, P. Variability in docking success rates due to dataset preparation. *J. Comput. Aided Mol. Des.* **26**, 775-786 (2012).
5. Huang da, W., Sherman, B. T. & Lempicki, R. A. Systematic and integrative analysis of large gene lists using DAVID bioinformatics resources. *Nat. Protoc.* **4**, 44-57 (2009).

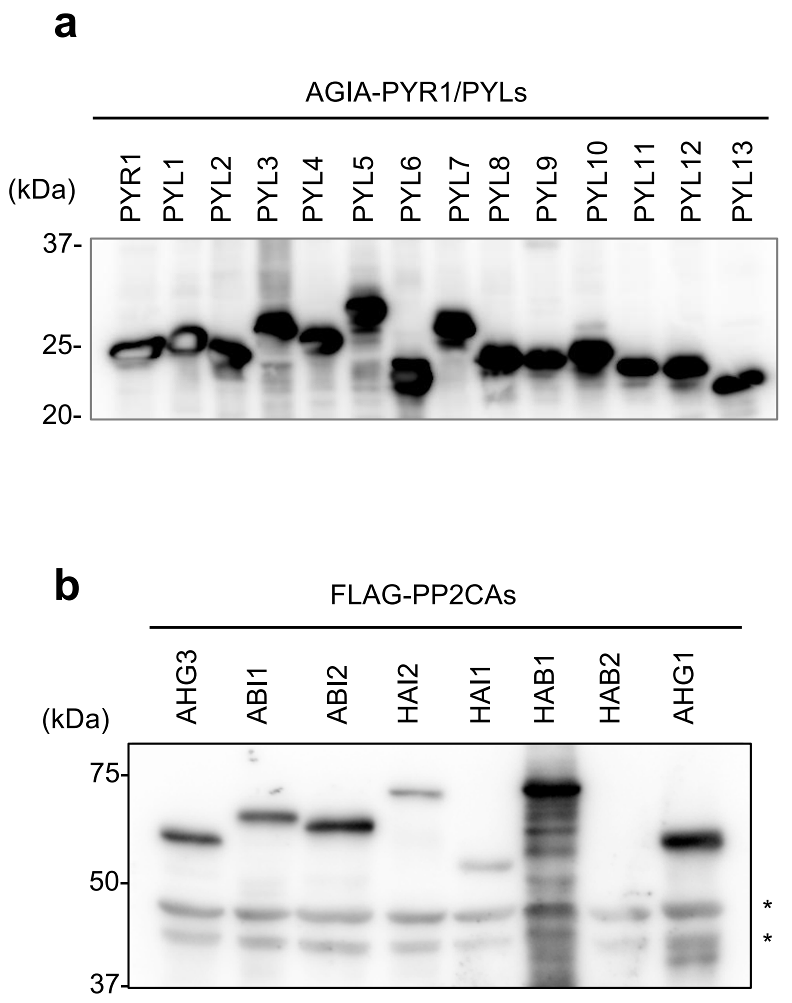

Supplementary Figure 1: Expression of recombinant ABA receptors and PP2CAs.

(**a**) Immunoblot analysis of AGIA-tagged PYR1 and PYL1-13. Expressed recombinant proteins were analyzed by immunoblotting anti-AGIA antibody. (**b**) Immunoblot analysis of recombinant N-terminal FLAG-tagged PP2CAs using anti-FLAG antibody. FLAG-HAB2 protein was not detected by immunoblot analysis. All recombinant proteins were expressed by a wheat cell-free system. The asterisk indicates a nonspecific band.

a

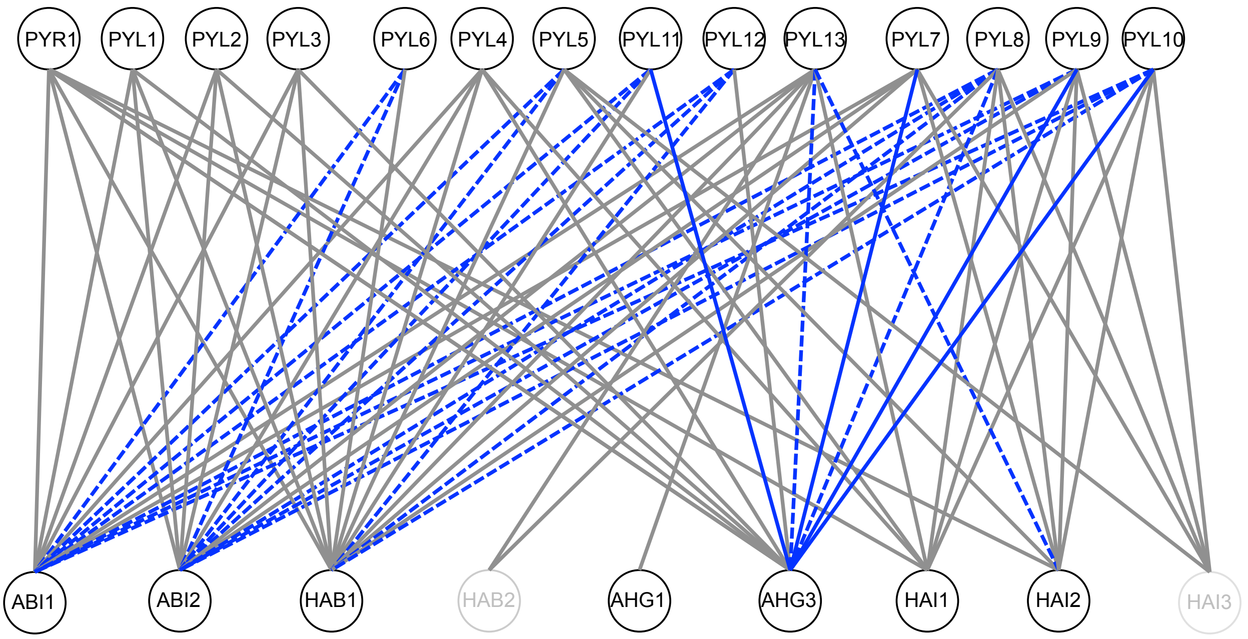

b

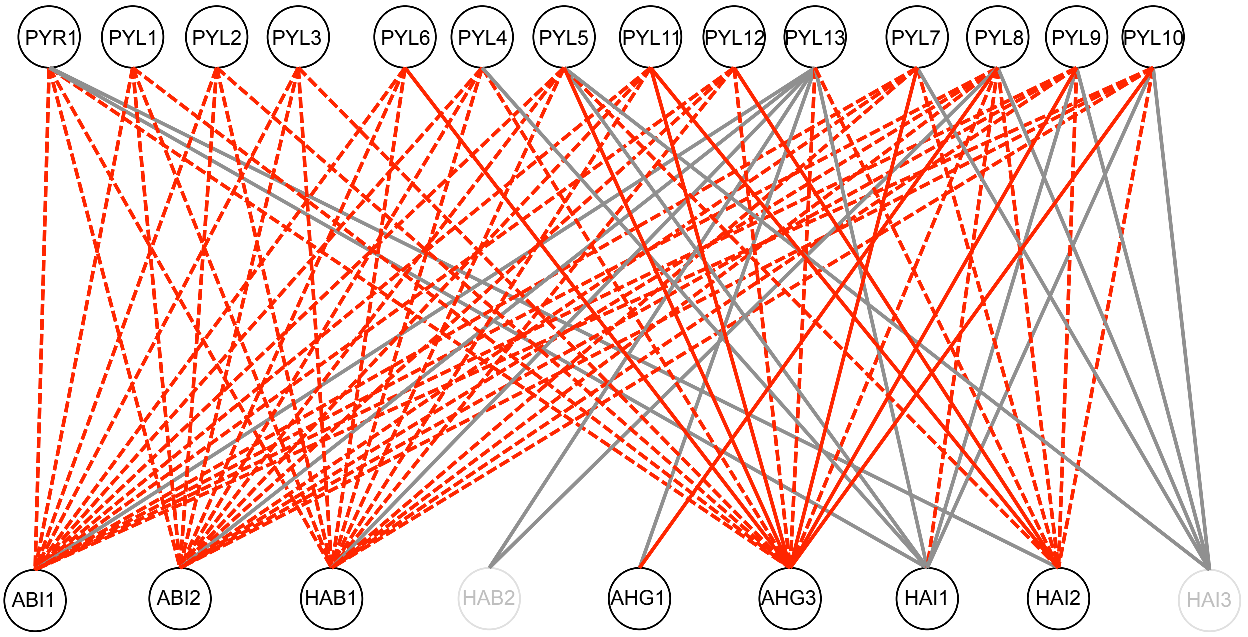

Supplementary Figure 2: Overview of interaction map of 14 ABA receptors and 7 PP2CAs. Interaction between 14 biotinylated ABA receptors and 7 FLAG-tagged PP2CAs in the absence (a) or presence of ABA (b). Blue lines, interactions in the absence of ABA; red lines, interactions in the presence of ABA; blue or red solid lines, interactions revealed in this study; blue or red dotted lines, interactions consistent with previous studies (BioGRID); grey lines, interactions has been reported in previous studies (BioGRID). HAB2 and HAI3 were not analyzed in this study.

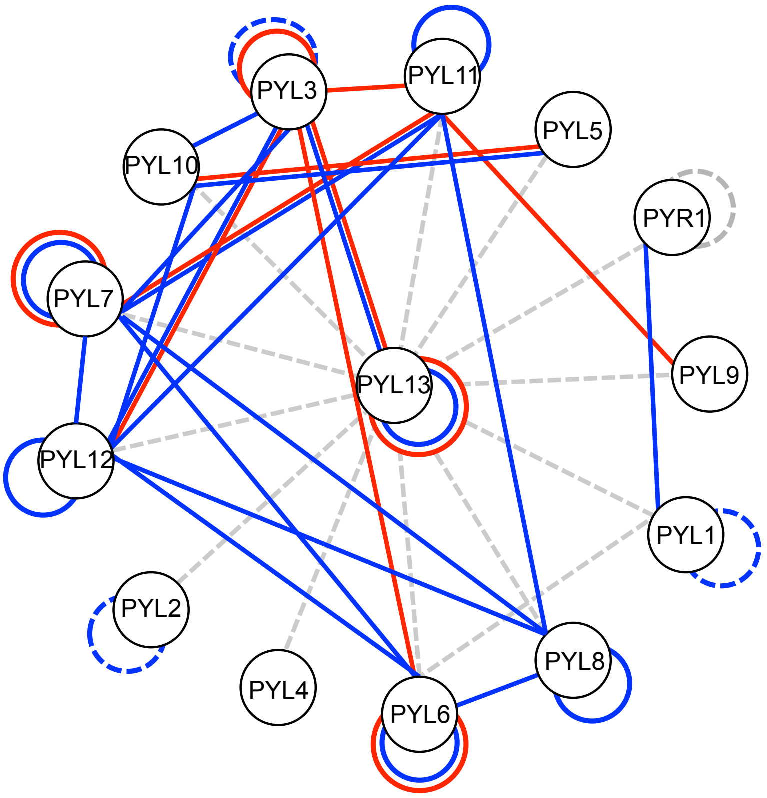

Supplementary Figure 3: Overview of interaction map of ABA receptors.

Interaction between 14 biotinylated ABA receptors and 14 AGIA-tagged ABA receptors in the absence or presence of ABA. Blue lines, interactions in the absence of ABA; red lines, interactions in the presence of ABA; blue or red solid lines, interactions revealed in this study; blue or red dotted lines, interactions consistent with previous studies (BioGRID); grey dotted lines, interactions has been reported in previous studies (BioGRID);

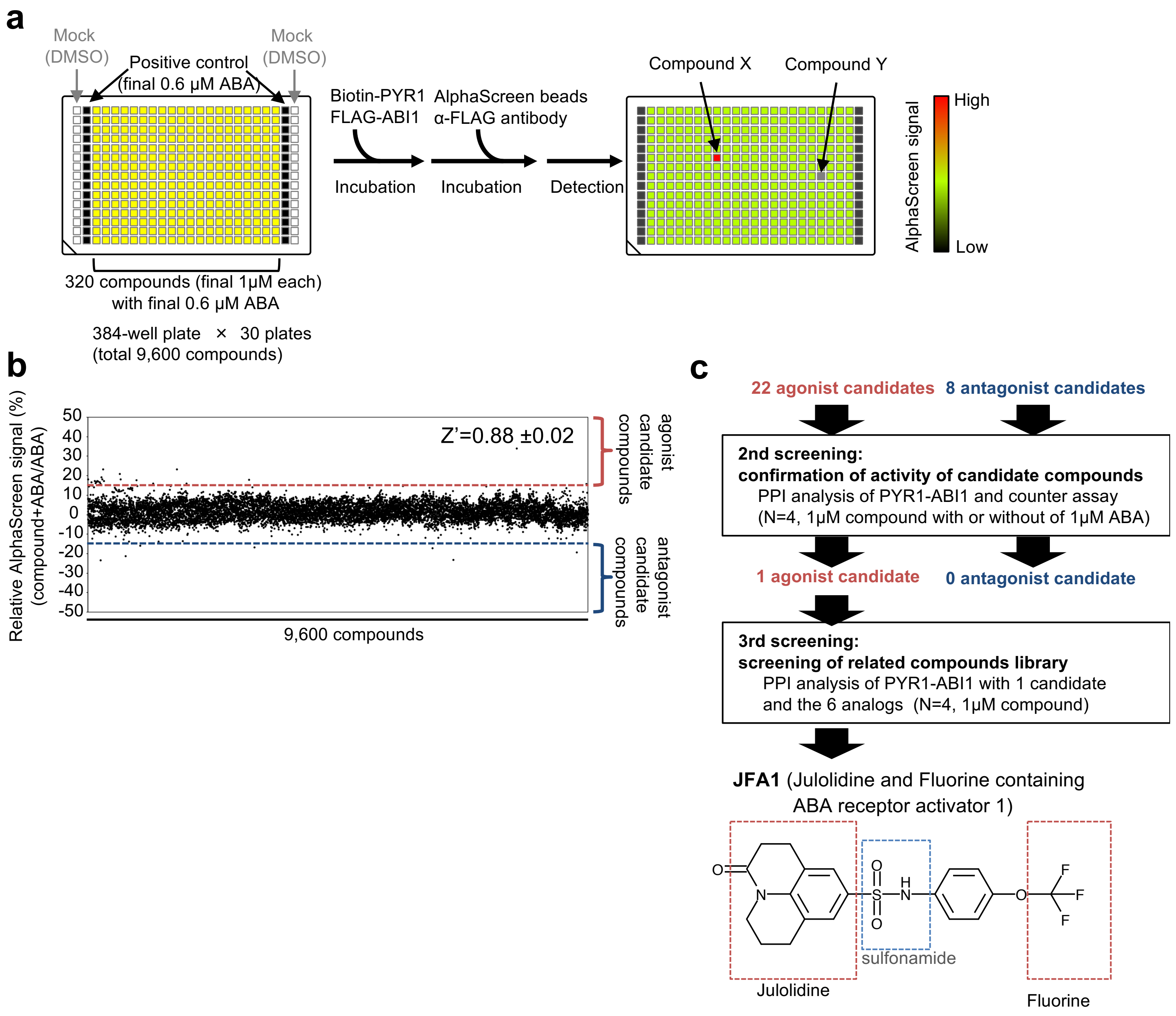

Supplementary Figure 4: Chemical compounds screening based on a wheat cell-free system.
(**a**) Over-view of 9,600 chemical compounds screening based on analysis of protein-protein interaction (PPI) between biotinylated PYR1 (biotin-PYR1) and FLAG-ABI1 (a detailed method is described in the method). (**b**) Result of 9,600 chemical compounds screening. Data were expressed as relative values normalized by the value of the AlphaScreen signal between biotinylated PYR1 and FLAG-ABI1 in the presence of 1μM ABA only. The broken line indicates 15 % enhancement (red) or inhibition (blue). (**c**) Flow chart of ABA agonist or antagonist candidates validation to identify compounds.

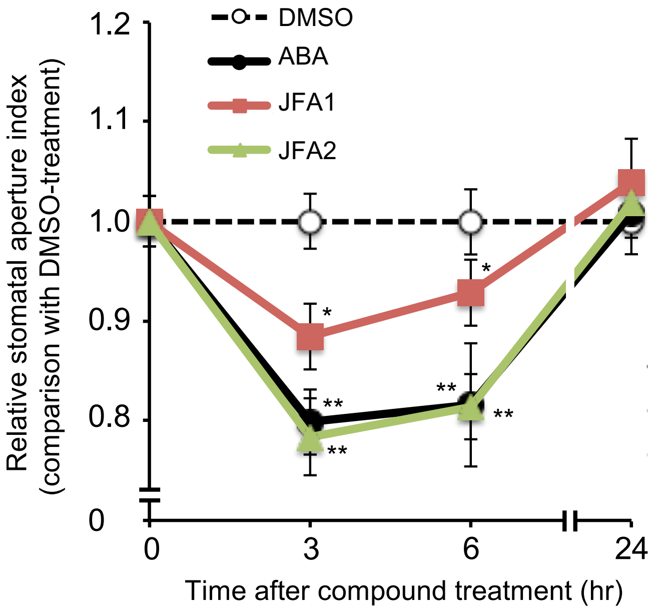

Supplementary Figure 5: Stomatal closure test in intact-plant.
Four-week old plants were sprayed with chemical solutions containing with 25 µM chemical, ABA, JFA1, or JFA2, and 0.02% Silwet L-77. Then plants were incubated in a growth cabinet at 22 ° C under a light condition. After incubation for 3, 6 and 24 hr, leaves were detached and treated with water containing with 1 µM rhodamine 6G (Sigma) for 2 min. Imaging of stomata was performed with a fluorescence microscope IX-73 (Olympus). Image analysis was performed using ImageJ software (https://imagej.nih.gov/ij/). The width and the length of the stomatal aperture were measured, and the stomatal aperture index was calculated by division of the aperture width through the length. Relative stomatal aperture index was expressed as a relative value with the DMSO-treatment as one. Stomatal aperture indexes are calculated from three independent experiments with 30 stomata per treatment. Error bars represent standard error. Statistically significant changes compared with mock control (DMSO) are indicated (*$P < 0.05$, **$P < 0.01$, two-tailed Student's t-test)

Figure 3d

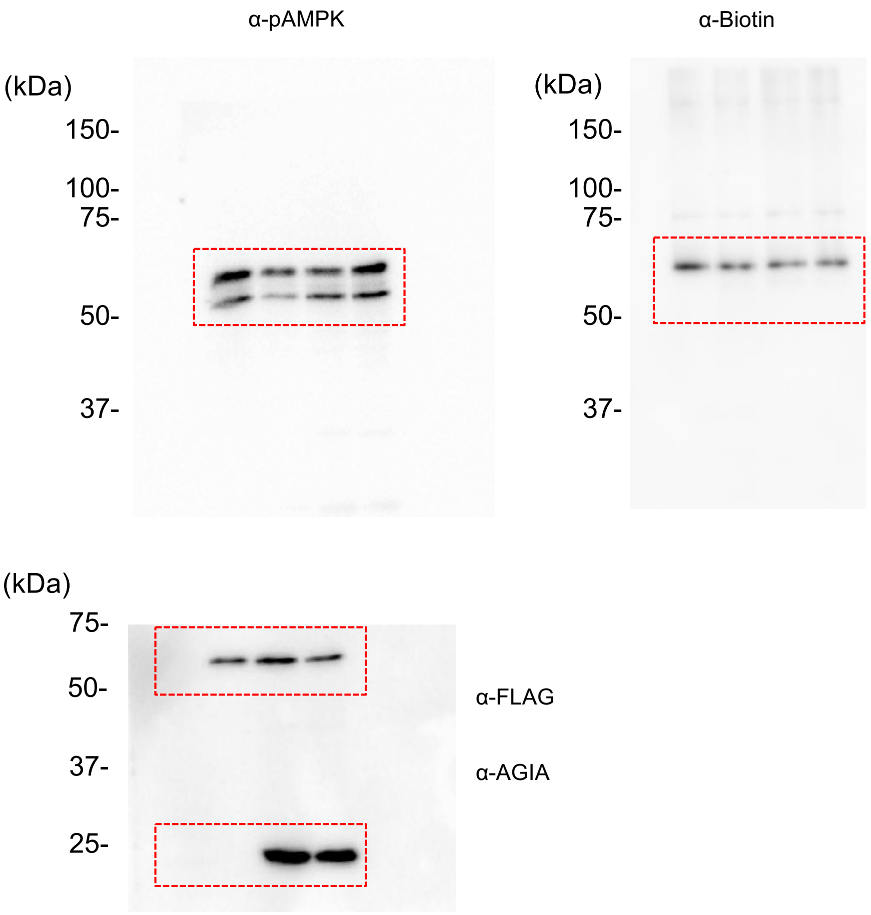

Supplementary Figure 1a

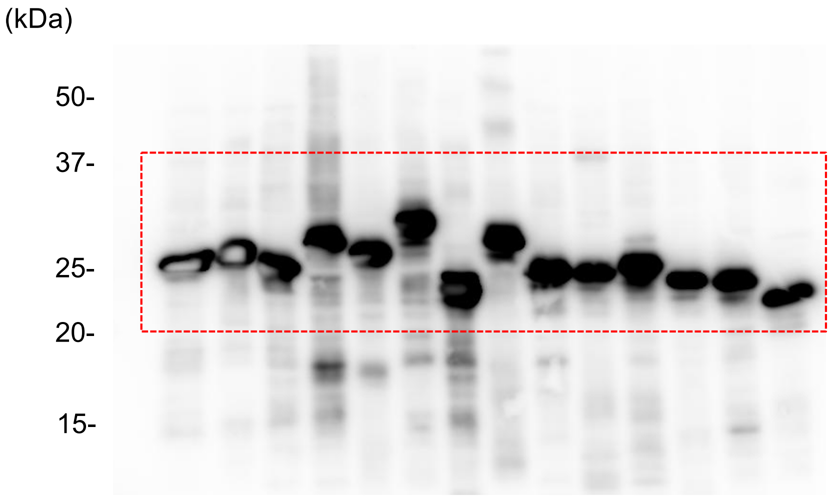

Supplementary Figure 1b

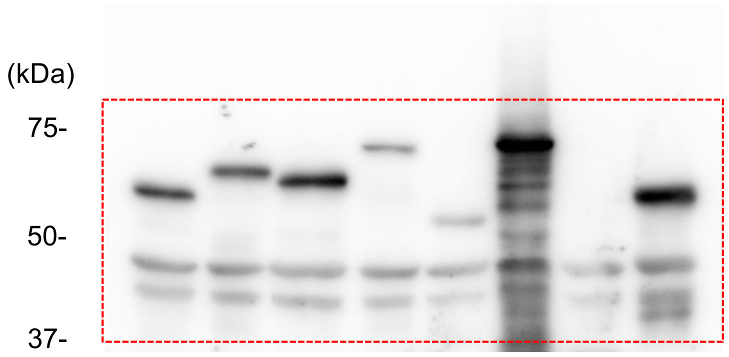

Supplementary Figure 6: : Uncropped blot images.

Supplementary Table 1. The best scores of docking simulations of PYR1 with JFA1, JFA2, and Qui. HM designates "Homology Model".

**Compounds**	**JFA1**		**JFA2**		**Qui**
**Docking modes/Template**	**HM**	**3NJO**	**HM**	**3NJO**	**HM**
**tether**	-9.6734	-10.1663	-11.0097	-9.1457	-12.4822
**free**	-12.8619	-9.5284	-11.9113	-10.9391	-13.6150

Supplementary Table 2: List of PCR primers

AGI	Gene name	Primer name	Primer sequence (5'-3')	Notes	use for
Cloning					
Forward primers					
At1g01360	PYL9	S1-PYL9	ccacccaccaccaccaATGGACGGCGTTGAAGGCGG	Lower case letters indicaties S1-linker sequence	cloning and split-primer PCR
At4g01026	PYL7	S1-PYL7	ccacccaccaccaccaATGGAGATGATCGGAGGAGA	Lower case letters indicaties S1-linker sequence	cloning and split-primer PCR
At5g53160	PYL8	S1-PYL8	ccacccaccaccaccaATGGAAGCTAACGGGATTGA	Lower case letters indicaties S1-linker sequence	cloning and split-primer PCR
At4g27920	PYL10	S1-PYL10	ccacccaccaccaccaATGAACGGTGACGAAACAAAG	Lower case letters indicaties S1-linker sequence	cloning and split-primer PCR
At5g45860	PYL11	S1-PYL11	ccacccaccaccaccaATGGAAACTTCTCAAAAATATC	Lower case letters indicaties S1-linker sequence	cloning and split-primer PCR
At5g45870	PYL12	S1-PYL12	ccacccaccaccaccaATGAAAACATCTCAAGAACA	Lower case letters indicaties S1-linker sequence	cloning and split-primer PCR
At4g18620	PYL13	S1-PYL13	ccacccaccaccaccaATGGAAAGTTCTAAGCAAAAACG	Lower case letters indicaties S1-linker sequence	cloning and split-primer PCR
At5g05440	PYL5	S1-PYL5	ccacccaccaccaccaATGAGGTCACCGGTGCAACTC	Lower case letters indicaties S1-linker sequence	cloning and split-primer PCR
At2g40330	PYL6	S1-PYL6	ccacccaccaccaccaATGGCTGATGTGCCGGAGCACG	Lower case letters indicaties S1-linker sequence	cloning and split-primer PCR
At2g38310	PYL4	S1-PYL4	ccacccaccaccaccaATGCTTGCCGTTCACCGTCC	Lower case letters indicaties S1-linker sequence	cloning and split-primer PCR
At4g17870	PYR1	S1-PYR1	ccacccaccaccaccaATGCCTTCGGAGTTAACACC	Lower case letters indicaties S1-linker sequence	cloning and split-primer PCR
At5g46790	PYL1	S1-PYL1	ccacccaccaccaccaATGCCTTCCGATTTAACTCA	Lower case letters indicaties S1-linker sequence	cloning and split-primer PCR
At1g73000	PYL3	S1-PYL3	ccacccaccaccaccaATGAATCTTGCTCCAATCCA	Lower case letters indicaties S1-linker sequence	cloning and split-primer PCR
At2g26040	PYL2	S1-PYL2	ccacccaccaccaccaATGAGCTCATCCCCGGCCGT	Lower case letters indicaties S1-linker sequence	cloning and split-primer PCR
		attB1-S1	GGGGACAAGTTTGTACAAAAAAGCAGGCTTccacccaccaccaccaatg	underline indicate S1-linker sequence	cloning
Reverse primer					
At1g01360	PYL9	T1-PYL9	tccagcactagctccagaTCACTGAGTAATGTCCTGAG	Lower case letters indicaties T1-linker sequence	cloning
At4g01026	PYL7	T1-PYL7	tccagcactagctccagaTCAAAGGTTGGTTTCTGTAT	Lower case letters indicaties T1-linker sequence	cloning
At5g53160	PYL8	T1-PYL8	tccagcactagctccagaTTAGACTCTCGATTCTGTCG	Lower case letters indicaties T1-linker sequence	cloning
At4g27920	PYL10	T1-PYL10	tccagcactagctccagaTCATATCTTCTTCTCCATAG	Lower case letters indicaties T1-linker sequence	cloning
At5g45860	PYL11	T1-PYL11	tccagcactagctccagaTTACAACTTTAGATGAGCCAC	Lower case letters indicaties T1-linker sequence	cloning
At5g45870	PYL12	T1-PYL12	tccagcactagctccagaTTAAGTGAGCTCCATCATCT	Lower case letters indicaties T1-linker sequence	cloning
At4g18620	PYL13	T1-PYL13	tccagcactagctccagaTTACTTCATCATTTTCTTTG	Lower case letters indicaties T1-linker sequence	cloning
At5g05440	PYL5	T1-PYL5	tccagcactagctccagaTTATTGCCGGTTGGTACTTC	Lower case letters indicaties T1-linker sequence	cloning
At2g40330	PYL6	T1-PYL6	tccagcactagctccagaTTACGAGAATTTAGAAGTGT	Lower case letters indicaties T1-linker sequence	cloning
At2g38310	PYL4	T1-PYL4	tccagcactagctccagaTCACAGAGACATCTTCTTCTTG	Lower case letters indicaties T1-linker sequence	cloning
At4g17870	PYR1	T1-PYR1	tccagcactagctccagaTCACGTCACCTGAGAACCAC	Lower case letters indicaties T1-linker sequence	cloning
At5g46790	PYL1	T1-PYL1	tccagcactagctccagaTTACCTAACCTGAGAAGAGT	Lower case letters indicaties T1-linker sequence	cloning
At1g73000	PYL3	T1-PYL3	tccagcactagctccagaTCAGGTCGGAGAAGCCGTGG	Lower case letters indicaties T1-linker sequence	cloning
At2g26040	PYL2	T1-PYL2	tccagcactagctccagaTTATTCATCATCATGCATAG	Lower case letters indicaties T1-linker sequence	cloning
		attB2-T1	GGGGACCACTTTGTACAAGAAAGCTGGGTCtccagcactagctccaga	underline indicate T1-linker sequence	cloning
Split-primer PCR					
Gene specific primers					
At5g51760	AHG1	S1-AHG1	ccacccaccaccaccaATGACTGAAATCTACAGAA	Lower case letters indicaties S1-linker sequence	split-primer PCR
At3g11410	AHG3	S1-AHG3	ccacccaccaccaccaATGGCTGGGATTTGTTGCG	Lower case letters indicaties S1-linker sequence	split-primer PCR
At5g59220	HAI1	S1-HAI1	ccacccaccaccaccaATGGCTGAGATTTGTTACG	Lower case letters indicaties S1-linker sequence	split-primer PCR
At1g07430	HAI2	S1-HAI2	ccacccaccaccaccaATGGCGGATATTTGTTATG	Lower case letters indicaties S1-linker sequence	split-primer PCR
At1g72770	HAB1	S1-HAB1	ccacccaccaccaccaATGGAGGAGATGACTCCCG	Lower case letters indicaties S1-linker sequence	split-primer PCR
At4g26080	ABI1	S1-ABI1	ccacccaccaccaccaATGGAGGAAGTATCTCCGG	Lower case letters indicaties S1-linker sequence	split-primer PCR
At5g57050	ABI2	S1-ABI2	ccacccaccaccaccaATGGACGAAGTTTCTCCTG	Lower case letters indicaties S1-linker sequence	split-primer PCR
At3g01090	SnRK1.1	S1-SnRK1.1	ccacccaccaccaccaATGTTCAAACGAGTAGATGA	Lower case letters indicaties S1-linker sequence	split-primer PCR
					split-primer PCR
Universal primers					split-primer PCR
		deSP6E02-Flag-S1	GGTGACACTATAGAACTCACCTATCTCTCTACACAAAACATTTCCCTACATACAACTT<br>TCAACTTCCTATTATGGACTACAAGGATGACGATGACAAGCTccacccaccaccaccaATG	Lower case letters indicaties S1-linker sequence, and green color indicate FLAG tag epitope	split-primer PCR
		deSP6E02-AGIA-S1	GGTGACACTATAGAACTCACCTATCTCTCTACACAAAACATTTCCCTACATACAACTT<br>TCAACTTCCTATTATGGAAGAAGCAGCTGGTATTGCTCGCCCATTGGCTccacccaccac<br>caccaATG	Lower case letters indicaties S1-linker sequence, and red color indicate AGIA tag epitope	split-primer PCR
		SPu	GCGTAGCATTTAGGTGACACT		split-primer PCR
		pDONR221 1stA4080	AGCGTCAGACCCCGTAGAAA		split-primer PCR
		pDONR221 2ndA4035	GTCAGACCCCGTAGAAAAGA		split-primer PCR
		AODA2306	AGCGTCAGACCCCGTAGAAA		split-primer PCR
		AODA2303	GTCAGACCCCGTAGAAAAGA		split-primer PCR
RT-qPCR					
At5g52300	RD29B	F-RD29B	TATGAATCCTCTGCCGTGAGAGGTG		RT-qPCR
		R-RD29B	ACACCACTGAGATAATCCGATCCT		RT-qPCR
At5g66400	RAB18	F-RAB18	GGCTTGGGAGGAATGCTT		RT-qPCR
		R-RAB18	TTGATCTTTTGTGTTATTCCCTTCT		RT-qPCR
At5g52310	RD29A	F-RD29A	GTTACTGATCCCACCAAAGAAGA		RT-qPCR
		R-RD29A	GGAGACTCATCAGTCACTTCCA		RT-qPCR
At5g59370	ACTIN4	F-ACTIN4	TACGGCTTTGGCTCCAAGTA		RT-qPCR
		R-ACTIN4	CTGCTTTCGCAATCCACAT		RT-qPCR

Supplementary Table 3: List of plasmids

AGI	Gene name	Plasmid backbones	expressed proteins
At1g01360	PYL9	pEU-His-bls-GW-STOP	His-biotin-PYL9
At4g01026	PYL7	pEU-His-bls-GW-STOP	His-biotin-PYL7
At5g53160	PYL8	pEU-His-bls-GW-STOP	His-biotin-PYL8
At4g27920	PYL10	pEU-His-bls-GW-STOP	His-biotin-PYL10
At5g45860	PYL11	pEU-His-bls-GW-STOP	His-biotin-PYL11
At5g45870	PYL12	pEU-His-bls-GW-STOP	His-biotin-PYL12
At4g18620	PYL13	pEU-His-bls-GW-STOP	His-biotin-PYL13
At5g05440	PYL5	pEU-His-bls-GW-STOP	His-biotin-PYL5
At2g40330	PYL6	pEU-His-bls-GW-STOP	His-biotin-PYL6
At2g38310	PYL4	pEU-His-bls-GW-STOP	His-biotin-PYL4
At4g17870	PYR1	pEU-His-bls-GW-STOP	His-biotin-PYR1
At5g46790	PYL1	pEU-His-bls-GW-STOP	His-biotin-PYL1
At1g73000	PYL3	pEU-His-bls-GW-STOP	His-biotin-PYL3
At2g26040	PYL2	pEU-His-bls-GW-STOP	His-biotin-PYL2
At1g01360	PYL9	pDONR221	-
At4g01026	PYL7	pDONR221	-
At5g53160	PYL8	pDONR221	-
At4g27920	PYL10	pDONR221	-
At5g45860	PYL11	pDONR221	-
At5g45870	PYL12	pDONR221	-
At4g18620	PYL13	pDONR221	-
At5g05440	PYL5	pDONR221	-
At2g40330	PYL6	pDONR221	-
At2g38310	PYL4	pDONR221	-
At4g17870	PYR1	pDONR221	-
At5g46790	PYL1	pDONR221	-
At1g73000	PYL3	pDONR221	-
At2g26040	PYL2	pDONR221	-
